# Quantitative discometry: Low-dose concordant pain onset identifies sensitized annular nociceptors under pressure–volume–controlled provocation

**DOI:** 10.1016/j.inpm.2026.100738

**Published:** 2026-01-30

**Authors:** Richard Derby, Yakov Vorobeychik

**Affiliations:** a Spinal Diagnostics and Treatment Center, Daly City, CA, 94015, USA; b Penn State Milton S. Hershey Medical Center, Penn State College of Medicine, Department of Anesthesiology and Perioperative Medicine, USA

**Keywords:** Discography, Discometry, Discogenic low back pain, Annular fissure, Vertebral endplate, Vertebrogenic pain, Intradiscal pressure, Internal disc disruption

## Abstract

**Background:**

Provocative discography is controversial because it couples mechanically induced disc stimulation to a subjective pain report and is often interpreted as a simple yes/no test of “discogenic pain.” Pressure–volume–controlled discography (“discometry”) allows disc provocation to be treated as a dose–response experiment using static pressure above opening (ΔP) and cumulative mechanical work (W), rather than pressure alone.

**Objective:**

To determine whether discs that reproduce a patient's concordant pain under pressure–volume control exhibit a distinct low-dose onset phenotype compared with discs that never declare, and to interpret these patterns in the context of annular versus vertebrogenic pain mechanisms.

**Methods:**

We retrospectively analyzed pressure–volume–controlled lumbar discography from a single outpatient spine practice. Discs were injected in fixed volume increments with static plateau pressures recorded at each step under protocol caps (ΔP ≤ 50 psi above opening; volume ≤3.5 mL). For each disc, we identified either the onset event (first 0.5 mL step with definite concordant pain ≥4/10, sustained ≥30 s) or a final-negative (censored) event if no onset occurred under the caps (i.e., the disc “never declared”). Static ΔP at the event (ΔP_event) and cumulative mechanical work (W_event = Σ ΔP × ΔV) were calculated, and an energy-equivalent stiffness (K_eq = 2W/V^2^) was derived. We compared onset versus censored doses across MRI morphologies (normal, fissured, disrupted), within encounters containing both positive and negative discs, and across apparatus conditions, and examined the volume step at which onset occurred.

**Results:**

Structurally abnormal discs that reproduced the presenting pain almost always did so at relatively low ΔP_event and W_event, yet with moderate-to-severe pain intensity at onset. Discs that never declared under the same protocol caps tolerated substantially higher ΔP_event and W_event, and morphologically normal discs clustered at the extreme of high-dose tolerance with rare positive responses. This separation between low-dose onset and high-dose tolerance persisted within fissured and disrupted strata, within encounters (positive vs negative discs in the same patient), and after stiffness normalization using K_eq, arguing against generalized “softness” as the sole explanation. In a subset with complete step-index data, most onset-positive discs declared by approximately 1.7 mL of injected volume and nearly all by 1.7–2.2 mL under slow, staged injection.

**Conclusion:**

These findings support a reproducible low-dose, high-intensity concordant pain phenotype in structurally abnormal discs, most consistent with chemically and mechanically sensitized nociceptors in the annulus and at the annulus–endplate junction. Other discs—whether morphologically normal or with more prominent vertebrogenic features—remain load-tolerant under bounded pressurization, suggesting different dominant generators. In this framework, pressure–controlled discography functions less as a binary test and more as a quantitative phenotyping tool that uses combined pressure and work thresholds to distinguish annular-/junctional-dominant low-dose responses from higher-dose, load-tolerant patterns, and may help refine patient selection for disc-directed versus vertebrogenic-targeted interventions in future prospective studies.

## 1 Introduction

Low back pain is common, costly, and heterogeneous. Among its plausible generators are the intervertebral disc and the vertebral endplate. Provocative discography—infusing contrast into an intervertebral disc while the awake patient reports pain—has long been used to investigate discogenic pain [[1], [2], [3]]. Its clinical utility remains debated, in part because the test combines a mechanical stimulus with a subjective pain report [4,5]. Two related concerns follow: specificity (does a “positive” response truly identify the disc as a relevant pain generator?) and mechanistic ambiguity (is the response driven by sensitized annular nociceptors, vertebrogenic/endplate loading, or iatrogenic over-distention of a degenerated structure?). In practice, these mechanisms are unlikely to be mutually exclusive: the annulus, annular–endplate junction, and adjacent vertebral marrow share overlapping nociceptive innervation via sinuvertebral and basivertebral pathways, such that disc provocation engages a blended anterior column nociceptive field rather than a single isolated tissue [[6], [7], [8]]. Extensive human and animal studies confirm that disc degeneration promotes sensory nerve ingrowth into the inner annulus, upregulation of cytokines such as TNF-α, IL-1β, IL-6, and NGF, and local instability — all of which increase nociceptor sensitization and support discogenic pain as a chemically and mechanically amplified process [9].

Pressure-controlled discography, whether performed manually or with automated pumps, seeks to improve interpretability by relating concordant pain to the pressure–volume dose under which it occurs. Early pressure-controlled experience, including a prospective discometry series reported in the Interventional Spinal Injection Society's (IPSIS) scientific bulletin, suggested that absolute compressive load was less important than the rise above opening pressure (ΔP, psi) for provoking concordant pain. In that series, static pressure–volume measurements were obtained in both side-lying and sitting positions and showed that concordant onset tended to occur at similar ΔP above opening even when intrinsic intradiscal pressure differed threefold between postures. Irritable or fissured discs typically produced concordant pain at relatively low ΔP (often on the order of ∼15 psi above opening), whereas other discs required higher ΔP (∼30 psi or more) before responding, and pain rarely required >50 psi above opening [10]. This preliminary work, together with a subsequent report building on the same dataset [11] and a later pressure-controlled classification analysis [12], was used to develop a pragmatic 0–4 pressure-based discogram scheme. That scheme distinguished low-ΔP “chemical” sensitization (pain ≥6/10 at <30 psi or <15 psi above opening) from higher-ΔP “mechanical” sensitization (pain ≥6/10 at 30–50 psi or ≥15 psi above opening) and was subsequently associated with different surgical outcome profiles [13]. Together, these early society-based and peer-reviewed studies provided preliminary physiologic support for above-opening pressure as a clinically relevant parameter and motivated the approximate 15–30–50 psi working thresholds that continue to frame contemporary pressure-controlled discography.

Subsequent peer-reviewed analyses began to define a physiologic baseline for these patterns. In a pressure-controlled study of asymptomatic volunteers, including both lay participants and experienced discographers who met the same asymptomatic criteria, no disc produced pain below ∼20 psi above opening, and even at ≥30–40 psi evoked pain was generally mild, regardless of MRI degeneration or Dallas grade-3 annular tears [14]. In a companion Spine Journal series restricted to grade-3 annular tears, negative discs in symptomatic patients closely resembled asymptomatic discs in both pressure–volume thresholds and low pain intensities, whereas positive discs deviated sharply, with lower onset pressures and volumes and markedly higher pain scores at matched pressure checkpoints [15].

These data argued that structural disruption alone does not distinguish painful from painless discs [16]; rather, symptomatic discs tend to cluster among those with outer annular disruption and low-dose sensitivity, whereas many structurally abnormal discs retain a mechanical tolerance profile similar to asymptomatic discs. Related dynamic manometry work by O'Neill, and colleagues, using a similar controlled-injection approach, further supported the notion that disc responses can be characterized by their pressure–response behavior and that symptomatic and asymptomatic discs occupy different regions of pressure–response space [17]. More recent vertebrogenic models have added that damaged endplates and adjacent marrow can also become pain sources, mediated through basivertebral pathways, inviting a more blended view of anterior column nociception in which annular and vertebral contributions often coexist [18,19].

Pressure–volume–controlled discography (“discometry”) offers a way to treat disc provocation more explicitly as a dose–response experiment within this overlapping nociceptive landscape. In our practice, infusion proceeds in fixed increments (typically an opening 0.2 mL step followed by 0.5 mL steps), with pressures recorded at static plateaus after dynamic transients dissipate. Two natural dose metrics follow: static pressure above opening (ΔP, psi) as an instantaneous load measure and mechanical work (W, psi·mL) as a cumulative dose, calculated as the sum of ΔP × ΔV across steps. Concordant pain onset is defined as the first step at which definite, sustained concordant pain is reported under preset safety caps (ΔP ≤ 50 psi; V ≤ 3.5 mL), while discs that never reach onset contribute a censored dose representing their tolerance under the same constraints. When low-dose, high-intensity concordant pain arises while contrast remains confined to the annulus or annular–endplate junction on lateral fluoroscopy, the pattern is most readily interpreted as activation of chemically sensitized annular/junctional nociceptors, and those regions become plausible targets for restorative strategies aimed at promoting structural healing and reducing peripheral sensitization.

Building directly on this lineage, the present discometry analysis extends prior pressure-controlled work by using these plateau-based ΔP and W metrics to treat disc provocation as a fully dose-resolved experiment. In contrast to earlier analyses that focused primarily on peak pressures or early thresholds, we systematically compare onset versus final-negative (censored) doses within and across MRI morphologies, incorporate stiffness-normalized checks, and retain negative discs as informative tolerance observations. Within this framework, pressure-controlled discography is not viewed as choosing between purely “annular” versus purely “vertebrogenic” pain, but to identify reproducible low-dose versus high-dose phenotypes that likely reflect different dominant nociceptive territories within a shared annular–endplate–vertebral complex. This design allows us to test whether low-dose responses, which are most consistent with sensitized annular and annular–endplate junctional nociception, reliably separate from the tolerance of non-declaring levels and whether those patterns are robust to morphology, apparatus conditions, and disc stiffness.

## 2 Methods

### 2.1 Study design and setting

This study is a retrospective disc-level analysis of prospectively collected pressure–volume–controlled lumbar discography (“discometry”) data from a single specialty spine practice (Spinal Diagnostics and Treatment Center) by a single interventionalist (RD). Consecutive discography encounters with recorded manometry, stepwise pain ratings, and MRI-based morphology were identified from an electronic database spanning routine clinical care between 1997 and 2015. Throughout this period, discography data were captured prospectively in a standardized fashion, with all pressures, injected volumes, and patient-reported responses recorded in real time during the procedure for subsequent entry into the database.

The unit of analysis for the present work is the intervertebral disc level, with a prespecified within-encounter subset used to compare positive and negative levels in the same patient. All procedures were performed by a single experienced interventional spine physician (R.D.), using a standardized Derby pressure-controlled discography protocol [3,20] This retrospective analysis of de-identified data was approved by the North Star Review Board (protocol NB500328; Derby_Discometry_IRB_Protocol_v2.1_2025-09-26), and all procedures were performed in accordance with the Declaration of Helsinki and institutional guidelines. The IRB granted a waiver of informed consent because the analysis used de-identified retrospective data.

Key terms and quantitative metrics used in this analysis are summarized in Appendix A (Glossary).

### 2.2 Encounter identification and inclusion

We queried the clinical database for all discography encounters in which at least one lumbar disc level was injected and manometry data were saved. Encounters were eligible for the source cohort if they met all of the following criteria:

• Lumbar discography performed using a two-needle technique with 3.5 in 23-gauge introducer through which a 6 in 25-gauge needle was directed into the intervertebral disc using a posterolateral approach under fluoroscopic guidance;
• Injection performed with the Derby manual pressure-controlled protocol, with recorded injection volumes and manometry recordings;
• Stepwise pain intensity and concordance assessments documented at each injection step on a 0–10 numeric scale; and
• Lumbar MRI or report available prior to the discogram, suitable for morphology classification.

We excluded encounters with cervical or thoracic discography only; levels including only dynamic recorded pressures, incomplete or corrupted manometry files; or missing pain documentation such that stepwise responses could not be interpreted. After applying these criteria, 330 lumbar discography encounters constituted the source encounter cohort.

### 2.3 Disc-level eligibility and morphology

Within eligible encounters, we evaluated all injected lumbar levels (L1–S1). A disc level was eligible for dose–response analysis if:

• The opening volume (typically 0.2 mL) and at least one subsequent 0.5 mL increment were recorded;
• Static pressure at opening and at the majority of post-opening steps could be obtained directly or reliably reconstructed from the contemporaneous manometry readings and recorded plateau pressure–volume series; and
• Stepwise pain ratings and concordance assessments were documented for each injected increment.

Across the full-time span, dynamic pressure recordings were available for almost all procedures, whereas explicit static plateau pressures at both low and higher ranges were routinely recorded in approximately 20–30 % of discograms, more commonly in earlier years. For the present analysis, we restricted to disc levels in which static plateau values could be reliably obtained or reconstructed for at least 70 % of post-opening 0.5 mL increments. This curated set of manually injected levels, referred to as the manual HILO subset (table disco_level_parts_manual_hilo_v2), provides the basis for all static ΔP and work metrics reported here.

Disc-level morphology for each eligible level was determined from a combination of pre-procedure MRI reports, intra-procedural fluoroscopic appearance at discography, and the operator's dictated description of disc disruption, fissuring, and endplate change. These text sources were extracted from the clinical database using custom regular-expression–based parsers and reconciled into a single categorical morphology field. Levels were categorized as:

• **Normal** – no significant degeneration, fissure, or endplate defect;
• **Fissured** – focal annular tear (e.g., Dallas Grade-3 radial fissure) without broad annular disruption [21];
• **Disrupted** – advanced degenerative change with broad annular disruption, vacuum phenomenon, and/or endplate damage; or
• **Other/unknown** – insufficient information to assign to the above categories.

The primary morphology analysis set consisted of levels classified as normal, fissured, or disrupted. Levels with unknown or ambiguous morphology were retained in the database and are reported descriptively but were not included in primary morphology-stratified comparisons.

Applying these eligibility criteria yielded 859 event-eligible lumbar disc levels from 364 discography encounters in the manual hi–lo table. Among these levels, 803 (93.5 %) had interpretable MRI morphology and were classified as normal (n = 229), fissured (n = 327), or disrupted (n = 247); the remaining 56 levels (6.5 %) had indeterminate or other morphology and were retained for descriptive analyses only (Table 1). The subsequent restrictions that define the final analytic cohort and its onset-positive subsets are summarized under Data selection and manual HILO pressure subset (Methods) and in Supplementary Table S1.

**Table 1:** Encounter and disc-level prevalence of onset vs censored events by morphology.

Morphology	n_total	n_onset (% within morph)	n_censor (% within morph)	% onset of all discs
Disrupted	247	197 (79.8 %)	50 (20.2 %)	22.9 %
Fissured	327	248 (75.8 %)	79 (24.2 %)	28.9 %
Normal (controls)	229	7 (3.1 %)	222 (96.9 %)	0.8 %

### 2.4 Discography protocol

Discography was performed as part of routine clinical care. Patients were positioned prone on a radiolucent table with standard monitoring. Local anesthetic was infiltrated along the planned needle trajectory to the dorsal fascia, avoiding intradiscal anesthetic administration. Minimal intravenous anxiolysis was used in most procedures, typically with low-dose midazolam (Versed) in small divided doses (generally ≤2 mg total), given to reduce anxiety while keeping patients fully awake and able to provide detailed pain reports. In a very small minority of cases on chronic high-dose opioids who were NPO for the procedure (estimated well under 1 % of encounters), low-dose meperidine (Demerol, generally 25–50 mg IV) was used to mitigate withdrawal-related hypersensitivity. On rare occasions, briefly titrated low doses of propofol (usually well under 100 mg total) were administered to facilitate needle placement in highly anxious or needle-intolerant patients, but any such medication was reduced or paused so that patients were fully responsive during pressure–volume testing and pain reporting. Lumbar discography was performed using a two-needle technique, utilizing a 25-gauge, 6-inch spinal needle directed through a 22 or 23-gauge, 3.5-inch introducer under biplanar fluoroscopic guidance. Multiple entries were avoided where possible and extraneous contrast injections that could alter pressure–volume behavior were minimized.

The injectate consisted of nonionic iodinated contrast (e.g., iohexol 240 mg I/mL) with pre-mixed antibiotic without added local anesthetic, steroid, or biologic agents. The contrast syringe was connected to the pressure transducer via low-compliance tubing as part of the pressure-controlled discography system.

All procedures followed a standardized Derby manual pressure–volume protocol [3,20]. After needle placement, a small volume of contrast (typically ∼0.2 mL) was injected slowly until the first clear rise in pressure, defining the opening pressure. Thereafter, 0.5 mL increments were injected at a controlled, slow rate of 0.125 mL/s. After each 0.5 mL increment, injection was paused to allow the pressure to stabilize at a static plateau, then the plateau pressure above opening was recorded and the patient was queried for pain intensity, quality, location, and concordance with their presenting pain.

Injections were mostly terminated at the earliest of: (1) static plateau ΔP ≥ 50 psi above opening; (2) total injected volume ≥3.5 mL; or (3) intolerable or clearly nonphysiological pain; or after concordant pain a 6/10 intensity or greater and one addition volume increment that validated the positive response. Dynamic pressure peaks during injection were not used for dose metrics; only static plateaus at each step were considered in the present analysis.

At each plateau, patients rated pain intensity on a 0–10 numeric rating scale. The operator documented intensity, quality (e.g., deep axial ache versus superficial or radicular pain), location, and whether the pain was familiar or concordant with the patient's typical symptoms. For this study, an onset-qualifying response was defined a priori as familiar or concordant pain with intensity ≥4/10, sustained for more than 30 s at the static plateau. In addition, if pain intensity was below 6/10, valid onset pain had to reach a level of 6/10 or greater concordant pain on subsequent volume increments.

### 2.5 Pressure measurement and data abstraction

The pressure–controlled discography system used a proximal pressure transducer (Merit Medical, Salt Lake City, UT) connected via a short, small-bore, low-compliance tubing segment (approximately 12 inches in length) to the contrast syringe. Intradiscal pressure was displayed continuously during injection, allowing dynamic injection peaks to be distinguished from static plateaus after injection ceased. Across the full cohort, manometry recordings were available for nearly all procedures; however, explicit static plateau readings at both low- and higher-pressure ranges were routinely documented in only a subset of discogram encounters. The manual HILO subset used in this analysis consists of levels in which static opening pressure and a majority of post-opening plateaus could be directly read or reliably reconstructed from the trace, following the plateau-based approach described in our earlier pressure-controlled discometry work [10]. Because any constant pressure offset across the tubing and needles would affect all steps equally within a given disc, our primary analyses focused on static plateau pressure above opening (ΔP) and on stepwise pressure differences between successive 0.5 mL volume increments when computing work, rather than on absolute pressure values.

The curated HILO table ('disco_level_parts_manual_hilo_v2') reconciles ambiguous or truncated opening readings by cross-checking neighboring steps and known apparatus behavior, then stores stepwise volumes, static and dynamic pressures, and pain responses for each 0.5 mL increment. This subset was chosen to minimize variability introduced by differing injector types or tubing configurations and to provide consistent, plateau-based ΔP measurements in the clinically relevant range (0–50 psi above opening).

During each procedure, a designated assistant recorded opening and plateau pressures, injected volumes, and the operator–patient dialogue on pain intensity and concordance onto a standardized worksheet as they were called out in real time. Immediately after the procedure, the operator (RD) dictated the results into the report, using the worksheet—on which each volume increment and its corresponding pressure and pain assessments had been recorded—to ensure accuracy; these dictated reports later fed the electronic database.

Discography and manometry data for this study were then abstracted from the original full-text reports and linked manometry tracings using custom Python scripts and regular-expression–based text parsing, with the extracted fields stored in a relational SQL database. These tools identified encounter-level information (dates, injected levels, injector type), disc-level injection sequences (step volumes and static plateau pressures), stepwise pain ratings and concordance judgments, and key imaging descriptors (including morphology and Modic status). To assess accuracy, a random subset of levels was manually checked against the original reports and manometry traces, confirming correct capture of opening pressure, stepwise volumes and plateaus, and pain ratings.

Disc levels with incomplete recording of doses or responses were uncommon. Such levels were retained in the extraction tables for descriptive purposes but were not used in event-based dose metrics if the a priori criteria for event definition (below) could not be applied. In the data abstraction, only pain explicitly described as concordant with the presenting symptoms in location and quality was coded as onset; pain described as nonspecific, atypical, or without clear concordance wording in the dictated report was treated as non-diagnostic, and such levels were retained as negative/censored.

### 2.6 Event definitions and dose metrics

#### 2.6.1 Onset events

For each analyzable level in the manual HILO subset, the injection sequence was summarized into a single event of one of two types: onset or final-negative (“censor”). An onset event was defined as the first 0.5 mL increment at which the awake patient reported definite concordant pain—reproducing their usual symptoms in both location and quality—with intensity ≥4/10, sustained for at least 30 s at the static plateau and occurring before the protocol safety caps were reached (ΔP ≤ 50 psi above opening and total injected volume ≤3.5 mL). Once onset criteria were met, subsequent increments did not redefine the onset dose, although all later plateaus and pain ratings were recorded for descriptive purposes and to determine whether the disc ultimately reached the ≥6/10 threshold for a clinically positive discogram.

#### 2.6.2 Final-negative (censor) events

Levels that never met onset criteria under the protocol safety caps were classified as negative. For these levels, the final injected step that remained within ΔP ≤ 50 psi above opening and total volume ≤3.5 mL was designated as the final-negative (censor) event, representing the highest tolerated static dose without concordant pain. Levels with missing or inconsistent documentation that prevented determination of onset status were uncommon; such levels remained in descriptive summaries but were not included in event-based dose comparisons.

#### 2.6.3 Static pressure above opening (ΔP_event)

For each event, the static pressure above opening was defined as

ΔP_event = P_plateau_event − P_opening,

where P_opening is the static plateau at the 0.2 mL opening step and P_plateau_event is the static plateau at the onset or censor step. ΔP_event therefore represents the incremental static pressure above opening at the event dose.

#### 2.6.4 Mechanical work (W_event)

Mechanical work up to the event (**W_event**) was treated as a cumulative dose metric and calculated as

W_event = Σ (ΔP_k × ΔV_k),

where **ΔV_k** is the volume increment at step *k* (typically 0.5 mL after opening) and **ΔP_k** is the static pressure above opening at that step, summed from the first post-opening increment through the event step. W_event captures both the magnitude and the accumulated exposure of static pressure above opening.

#### 2.6.5 Energy-equivalent stiffness (K_eq)

To approximate a stiffness-like property at the event dose, an energy-equivalent stiffness metric was calculated as:

K_eq = 2 × W_event / V_event^2^

where V_event is the total injected volume at the event step (including the opening step). Stepwise ΔP and W values were computed in dedicated mechanics roll-up tables and aggregated to event-level metrics in an event table (e.g., dxmh__keq_onset_censor_v3), which also included MRI morphology and encounter identifiers.

### 2.7 Data selection and manual HILO subset

Parsed discography data were stored in linked encounter-, level-, and level-part tables (disco_encounters, disco_level, disco_level_parts). The starting registry comprised 5812 Derby manual device pressure-controlled discogram disc levels across 2183 encounters (Supplementary Table S1, step 1).

Levels were first restricted to those included in the manual HILO table, yielding an event-eligible HILO cohort of 859 lumbar levels from 364 encounters (step 2). Within this event-eligible cohort, 698 levels (330 encounters) had static plateau pressures documented or reliably reconstructed for at least 70 % of recorded volume steps and were considered manual HILO levels with adequate parts data (step 3).

Additional inclusion criteria were applied—limiting analytic events to lumbar levels in which static plateau doses remained within ΔP ≤ 50 psi above opening and volume ≤3.5 mL, with adequate morphology/imaging data and overall data quality—to define the final analytic cohort used in the ΔP/W and K_eq analyses (477 levels; step 4). Within this cohort, 358 levels (75.1 %) were onset-positive under our operational criteria and formed the basis for onset versus censored dose comparisons (step 5). In earlier years, occasional injections—particularly in morphologically normal discs—transiently exceeded these values, but those higher-pressure steps were not used to define onset or censor doses in the present analysis.

Among these onset-positive levels, 196 (41.1 % of the analytic cohort; 54.7 % of onset-positive levels) had complete pain-intensity data for onset-intensity analyses. A secondary subset of 153 onset-positive levels from 128 encounters (128 patients) had complete onset step-index data for the step-index analyses (steps 6–7; see Section 3.3, Supplementary Fig. S1, and Supplementary Table S1).

### 2.8 Dose binning and quadrant definitions

To relate onset and censor events to clinically meaningful dose thresholds, we defined two complementary binning schemes.

For the primary “protocol 20/20” scheme, events were classified by ΔP and W as follows:

• ΔP rungs (psi above opening): 10–<20, 20–<30, and ≥30
• Work bands (psi·mL): <20, 20–<40, and ≥40

Crossing these rungs and bands yields a pressure–work grid. For graphical summaries and odds-ratio calculations, we aggregated these cells into four quadrants:

• **Q1:** low-ΔP/low-W
• **Q2:** low-ΔP/higher-W
• **Q3:** higher-ΔP/low-W
• **Q4:** high-ΔP/high-W

Event-level data and quadrant assignments were exported to a plot-ready table (e.g., dxmh__dose_plot_export_v1) used to generate stacked 100 % bar plots for onset versus censor events.

To align with historical “chemical” versus “mechanical” sensitivity thresholds, we also applied a 15/7.5 binning scheme that classified events according to ΔP <15 versus ≥15 psi and W < 7.5 versus ≥7.5 psi·mL. The resulting four “historical” quadrants were exported to a parallel table (e.g., dxmh__dose_plot_export_15_7p5_v1) for sensitivity analyses. We note that the 15 psi plateau threshold derives from earlier “chemical disc” descriptions, whereas the 7.5 psi·mL work cutoff was chosen pragmatically as a conservative low-work band that pairs with 15 psi; it does not represent an independent historical norm.

Within fissured and disrupted discs, energy-equivalent stiffness values (K_eq) were grouped into tertiles (e.g., dxmh__keq_morph_tertiles_v1) to examine whether onset–censor separation in ΔP_event persisted across increasing stiffness. These tertiles were used to plot ΔP_event medians and interquartile ranges (IQRs) for onset and censor events across K_eq strata.

### 2.9 Statistical analysis

All analyses were conducted at the disc level, with a predefined within-encounter subset for paired comparisons. We summarized counts and proportions of onset versus censor events by morphology (normal, fissured, disrupted), as well as medians and interquartile ranges of ΔP_event and W_event for onset and censor events within each morphology. Unknown or other morphologies were tabulated but not included in primary morphology-stratified comparisons.

Within each morphology category, we compared ΔP_event and W_event between onset and censor events using nonparametric tests (e.g., Wilcoxon rank-sum), given the skewed distributions of dose metrics. For encounters with at least one positive and one negative level, we performed within-patient paired comparisons of ΔP_event and W_event using paired nonparametric tests (e.g., Wilcoxon signed-rank) to assess whether, within the same clinical and apparatus context, positive levels generally declared at lower doses than negative levels.

To quantify how strongly onset and censor events occupied low-versus high-dose regions, we constructed 2 × 2 tables contrasting Q1 (low-ΔP/low-W) with Q4 (high-ΔP/high-W) and onset with censor events. Odds ratios (ORs) and 95 % confidence intervals were calculated using Fisher's exact test for the overall cohort and for the pooled fissured + disrupted subset. These ORs correspond to the stacked 100 % bar plots generated from the quadrant exports.

Primary comparisons of onset versus censored doses were conducted at the disc level and therefore do not fully account for clustering of discs within patients. To mitigate this, we complemented disc-level analyses with encounter-level paired comparisons (Section 3.6), which summarize doses within each encounter and directly compare positive and negative discs in the same patient.

Within fissured and disrupted discs, we examined whether onset–censor separation in ΔP_event persisted across K_eq tertiles by plotting ΔP_event medians and IQRs across tertiles for onset and censor events and by using rank-based methods to test for differences across stiffness strata. The goal was to evaluate whether low-dose onset in sensitized discs could be explained by generalized “softness” or whether it persisted after approximate stiffness normalization.

To test robustness to apparatus-related influences, we repeated key comparisons of ΔP_event, W_event, and Q1 versus Q4 odds ratios in a predefined low apparatus-volume-fraction subset, in which tubing and needle volume were small relative to injected volume and pressure losses across the system were expected to be minimal.

All statistical tests were two-sided. Given the physiologic and exploratory nature of the analyses, p-values are reported without formal adjustment for multiple comparisons, and interpretation emphasizes effect sizes, consistency across strata, and concordance with prespecified hypotheses rather than strict dichotomization by p-value thresholds. Statistical analyses and plotting were performed using Python (version 3.11) with standard scientific libraries (e.g., pandas, NumPy, SciPy, and matplotlib). Given the exploratory physiologic nature and the modest number of discs per patient, we judged these paired encounter-level summaries more appropriate than attempting complex mixed-effects modeling.

### 2.10 Human subjects and IRB approval

This retrospective observational study is reported in accordance with the STROBE guidelines for observational studies. This analysis of dictated clinical records from Spinal Diagnostics and Treatment Center (1997–2020) was reviewed and approved by the North Star Review Board (NSRB), an independent 501(c)(3) IRB (protocol NB500328; Derby_Discometry_IRB_Protocol_v2.1_2025-09-26). The study was determined to involve no more than minimal risk and was reviewed under the Common Rule via expedited Category 5 procedures (retrospective use of data collected for non-research purposes). Minors were excluded from participation. Informed consent was waived in accordance with 45 CFR 46.116(f)(3) because the research could not practicably be conducted without the waiver and all data were de-identified prior to analysis.

## 3 Results

### 3.1 Study sample and disc-level prevalence

The event-eligible HILO cohort comprised 859 lumbar disc levels from 364 discography encounters in the manual hi–lo table. At the disc level, 475 levels (55 %) reached concordant pain onset under the protocol caps, whereas 384 levels (45 %) remained negative and contributed a final-negative (censored) dose. Morphology was classifiable in 803 levels (93.5 %), distributed as 229 morphologically normal controls, 327 fissured discs, and 247 disrupted discs, with the remainder categorized as unknown (Table 1). Within this event-eligible set, 477 levels met all criteria for the final analytic cohort used in the ΔP/W and K_eq analyses (Supplementary Table S1).

Positive responses were rare in morphologically normal discs but common in structurally abnormal discs. Only a small minority of normal discs reached onset, whereas most disrupted and fissured discs declared positive under pressure–volume control. These prevalences reproduce the classic discography pattern of low positive rates in normal discs and high positive rates in fissured and disrupted discs, now expressed in a dose-resolved framework (Table 1).

At the encounter level, 267 of 364 discography encounters (73 %) contained at least one positive disc, while 97 encounters (27 %) contained only censored levels, indicating that discography is not globally positive across all levels. Most encounters with positive discs also included one or more negative discs, providing internal controls for comparing onset doses with the tolerance doses of discs that remained negative in the same patient and session. Fig. 4 illustrates a representative discogram in which adjacent negative levels serve as internal controls for a disc that declares abruptly at low volume and relatively low ΔP with high-intensity, concordant pain. We therefore next examined how the doses at which discs declared—expressed as static pressure above opening and cumulative work—differed between onset and censor steps by morphology (Section 3.2).

For prevalence the ‘onset’ includes discs with any concordant pain under the safety caps, regardless of intensity; the stricter ≥4/10 onset and ≥6/10 peak criteria used in the dose–response analyses are described in the Methods.

### 3.2 ΔP and work at onset vs final-negative by morphology

Static pressure above opening (ΔP_event) and cumulative work (W_event) differed markedly between concordant pain onset and final-negative doses within each MRI morphology category (Table 2). ΔP_event and W_event could be estimated at the event step for 803 levels with interpretable morphology, including some levels with less-complete plateau coverage that were not used in the more stringent quadrant and energy-equivalent stiffness (K_eq) analyses of the 477-level analytic cohort. ΔP_event was defined as the static plateau pressure above opening (plateau minus opening pressure) at the event step, and W_event as the cumulative sum of ΔP_step × ΔV_step across 0.5 mL increments up to the event. All values were derived from static plateaus; dynamic traces were used only for quality assurance (see Table 3).

**Table 2:** Onset and final-negative (censored) doses by morphology: median (IQR) ΔP_event and W_event from static plateaus.

Morphology	Event type	ΔP_event, psi median [IQR]	W_event, psi·mL median [IQR]	n
Disrupted	Onset	15.10 [9.55–24.45]	8.90 [3.38–20.15]	197
Disrupted	Final-negative (censored)	35.70 [26.10–49.90]	48.03 [25.76–61.13]	50
Fissured	Onset	16.50 [11.15–28.05]	8.16 [3.84–19.88]	248
Fissured	Final-negative (censored)	33.50 [16.85–58.25]	36.12 [16.40–61.77]	79
Normal (controls)	Onset	62.80 [61.15–77.80]	43.74 [41.98–73.75]	7
Normal (controls)	Final-negative (censored)	68.50 [57.50–74.90]	62.08 [47.98–77.44]	222

**Table 3:** Within-encounter paired comparison of onset vs final-negative doses.

Metric	n encounters	Onset discs^a^	Censored discs^a^	p (paired Wilcoxon)
ΔP_event (psi), level-wise	151	median 15.9	median 32.0	–
W_event (psi·mL), level-wise	151	median 9.64	median 23.6	–
ΔP_event (psi), encounter-level†	151	median 16.2	median 30.9	≈1.4 × 10^−14^
W_event (psi·mL), encounter-level†	151	median 9.86	median 24.4	≈3.1 × 10^−11^

a Values are medians across levels or encounters as indicated. †Encounter-level summaries use, within each encounter, the median ΔP_event or W_event across positive discs and across negative discs, then compare those paired medians across encounters.

In disrupted discs, concordant pain onset occurred at relatively low pressures and modest work, whereas censored doses required substantially higher values. Median ΔP_event at onset was 15.10 psi (interquartile range [IQR] 9.55–24.45) compared with 35.70 psi (IQR 26.10–49.90) at the final-negative step. Corresponding W_event values were 8.90 psi·mL (IQR 3.38–20.15) at onset and 48.03 psi·mL (IQR 25.76–61.13) at censor (n = 197 onset events, n = 50 censored events).

Fissured discs showed a similar pattern. Median ΔP_event at onset was 16.50 psi (IQR 11.15–28.05) versus 33.50 psi (IQR 16.85–58.25) at the final-negative step, and median W_event increased from 8.16 psi·mL (IQR 3.84–19.88) at onset to 36.12 psi·mL (IQR 16.40–61.77) at censor (n = 248 onset events, n = 79 censored events). Thus, in both fissured and disrupted discs, censored doses typically required roughly a two-fold or greater increase in ΔP and a four-to five-fold increase in W compared with the doses that first provoked concordant pain.

In contrast, morphologically normal control discs rarely reached onset criteria and tolerated high doses without reproducing the presenting pain. When onset occurred in normals, it did so only at very high pressures and work: median ΔP_event at onset was 62.80 psi (IQR 61.15–77.80) with median W_event 43.74 psi·mL (IQR 41.98–73.75), based on 7 positive levels. Their censored doses reached similarly high static loads—median ΔP_event 68.50 psi (IQR 57.50–74.90) and median W_event 62.08 psi·mL (IQR 47.98–77.44) across 222 negative levels. Taken together, these data show that within fissured and disrupted morphologies, concordant pain onset typically emerges at low ΔP and low W, whereas discs that never declare tolerate substantially higher ΔP and W before reaching the protocol safety caps, while normal discs sit at the opposite extreme with both onset (when present) and censor doses clustered at very high ΔP and W.

Definitions: ΔP_event is static pressure above opening at the event step; W_event is the cumulative Σ(ΔP_step × ΔV_step) to that step.

### 3.3 Intensity and abruptness of concordant pain onset

We next asked whether concordant pain at the onset step was typically marginal or clearly “clinically positive,” and whether it emerged abruptly or only after multiple dose escalations. This analysis used the same onset definition as in the ΔP and work comparisons—namely, the first 0.5 mL step with definite concordant pain meeting the ≥4/10 onset, ≥6/10 eventual peak, and ≥30-s duration criteria under the protocol safety caps (ΔP ≤ 50 psi; V ≤ 3.5 mL)—and contrasted these onset-positive levels with the censor (final-negative) steps described in Sections 3.1–3.2.

Among the 358 onset-positive levels with complete pain-intensity ratings that satisfied the ≥4/10 onset and ≥6/10 peak criteria, concordant pain intensity at onset was almost always moderate to severe (Fig. 1A). Only 2/358 (0.6 %) onsets occurred at 2/10, 6/358 (1.7 %) at 3/10, and 7/358 (2.0 %) at 4/10, yielding just 4.2 % of onsets at ≤4/10. In contrast, 23/358 (6.4 %) occurred at 5/10, and 320/358 (89.4 %) at 6–10/10. Overall, 343/358 (95.8 %) of onsets reached at least 5/10, and 255/358 (71.2 %) were ≥7/10. The median onset intensity was 7/10 (interquartile range [IQR] 6–8). Thus, once concordant pain appeared under pressure–volume-controlled injection, it almost always met or exceeded conventional thresholds for a clearly positive discogram rather than representing vague low-grade discomfort.

**Fig. 1A:**
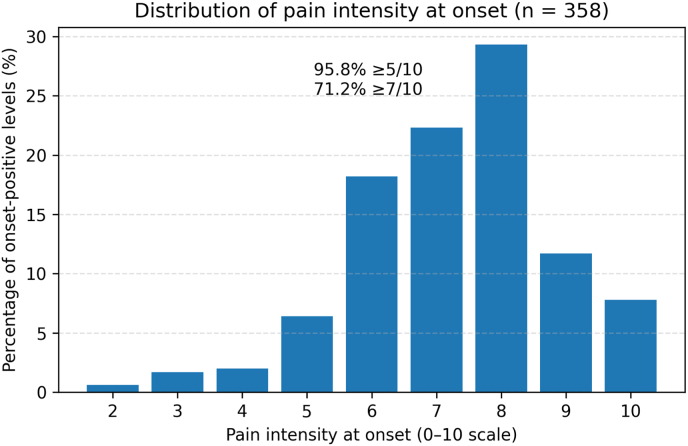
Distribution of pain intensity at onset Distribution of concordant pain intensity at onset (0–10 scale) within each morphology among onset-positive levels (n = 144 disrupted, 188 fissured, 5 normal, 21 unknown). Bars represent the percentage of onsets at each intensity bin; horizontal markers indicate median intensity for each morphology. In both disrupted and fissured discs, onset pain clustered in the 6–8/10 range (median 7/10), with only ∼4–5 % of onsets occurring at ≤4/10 and the majority ≥7/10. Morphologically normal discs were infrequently positive but, when positive, also showed high-intensity onset pain (median 8/10).

This pattern was consistent across morphologic categories (Fig. 1B). In disrupted discs (n = 144), onset intensities clustered in the 6–8/10 range (median 7/10), with only ∼5 % of onsets ≤4/10 and approximately two-thirds ≥7/10. Fissured discs (n = 188) showed a similar distribution (median 7/10, with peaks at 7–8/10 and roughly three-quarters of onsets ≥7/10). The small normal subset labeled positive (n = 5) also showed high intensities (median 8/10; no onsets ≤4/10), and unknown-morphology levels (n = 21) again behaved similarly (median 7/10; the majority ≥7/10). Companion analyses of the step index at which onset first occurred showed that concordant pain generally appeared after only a small number of additional 0.5 mL increments once the disc had reached the low-pressure safety range, with most onsets occurring at step indices 1–2 and only a minority requiring later steps (Supplementary Fig. S1). Taken together with the ΔP and work results, these findings are more consistent with a threshold event than with slow escalation: once onset occurs, pain intensity jumps quickly to clinically positive levels, whereas censor steps in the same encounters tolerate higher ΔP and W without ever reaching comparable pain.

**Fig. 1B:**
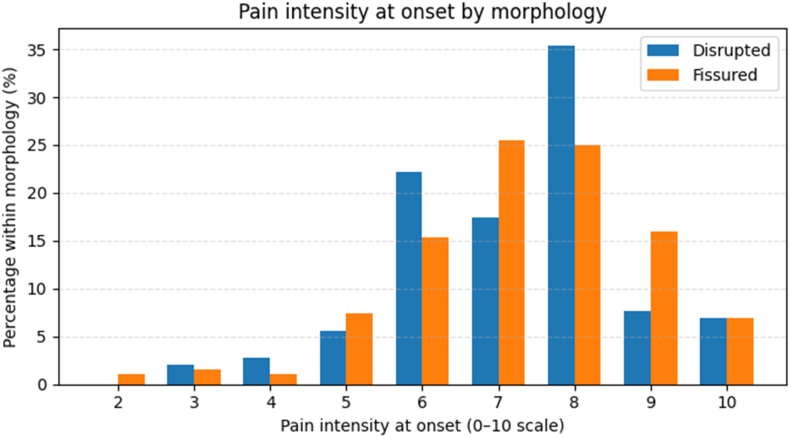
Pain intensity at onset by morphology: Prevalence of concordant pain onset versus final-negative (censored) events by MRI morphology. Bars show, for each category (disrupted, fissured, normal controls, unknown), the percentage of disc levels that reached onset under the protocol caps (ΔP ≤ 50 psi; V ≤ 3.5 mL) versus those that remained negative and contributed a censored dose. As in classic discography series, morphologically normal discs were rarely positive (7/229, 3.1 %), whereas onset was common in structurally abnormal discs (197/247 disrupted [79.8 %], 248/327 fissured [75.8 %]). Together, these panels show that abnormal discs are both more likely to declare and, when they do, typically produce robust, clinically positive pain at low pressure–volume doses.

### 3.4 ΔP × Work quadrants: onset vs final-negative

**Fig. 2:**
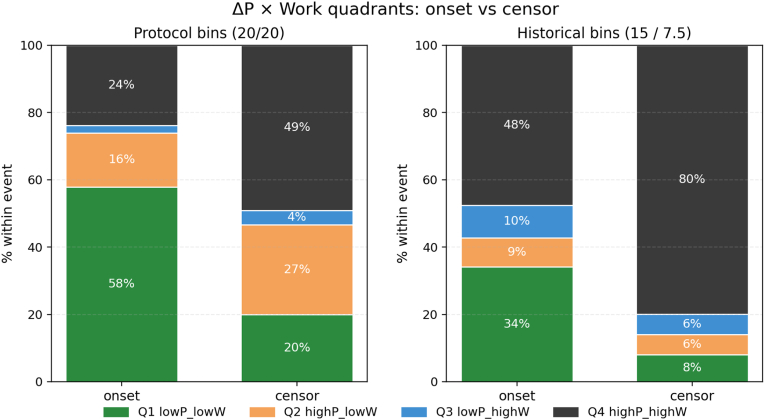
ΔP × Work quadrants for onset versus final-negative (censored) events. Left panel: protocol 20/20 grid (ΔP rungs 10–<20, 20–<30, ≥30 psi; work bands <20, 20–<40, ≥40 psi·mL). Right panel: historical 15/7.5 grid (ΔP <15 vs ≥ 15 psi; work <7.5 vs ≥ 7.5 psi·mL). Bars show the proportion of events within each quadrant for onset and censored levels; labels indicate percentages within event type. Under both binning schemes, onset events concentrate in the low-ΔP/low-W quadrant (Q1), whereas censored events cluster in the high-ΔP/high-W quadrant (Q4).

To visualize the joint distribution of static pressure above opening and cumulative work at the event dose, we classified each disc level with complete ΔP and W measurements into ΔP × Work “quadrants” under two prespecified binning schemes (Fig. 2). The protocol 20/20 grid used ΔP rungs of 10–<20, 20–<30, and ≥30 psi, and work bands of <20, 20–<40, and ≥40 psi·mL. The historical 15/7.5 grid applied prior “chemical” versus “mechanical” sensitivity thresholds using ΔP <15 versus ≥15 psi and work <7.5 versus ≥7.5 psi·mL. Within each scheme, Q1 denotes low-ΔP/low-W, Q2 low-ΔP/high-W, Q3 high-ΔP/low-W, and Q4 high-ΔP/high-W.

Under the protocol 20/20 grid (Fig. 2, left panel), onset events were strongly enriched in the low-dose quadrant, whereas censored events clustered in the high-dose quadrant. Among 311 onset events, 58 % fell in Q1 (low-ΔP/low-W), 16 % in Q2, 2 % in Q3, and 24 % in Q4. In contrast, among 532 censored events, only 20 % were in Q1, 27 % in Q2, 4 % in Q3, and 49 % in Q4. Thus, nearly three-fifths of onsets occurred at simultaneously low ΔP and low W, whereas roughly half of censored events required both high pressure and high work.

Collapsing the grid to a 2 × 2 comparison of Q1 versus Q4 yielded a strong separation of onset and censored events. Overall, the odds that a Q1 event was an onset rather than a censor were 5.99 (95 % confidence interval [CI] 4.21–8.52; Fisher's exact p ≈ 2.3 × 10^−12^). When restricted to fissured and disrupted discs, the enrichment remained robust with an odds ratio of 4.05 (95 % CI 2.74–5.98; p ≈ 1.2 × 10^−12^).

The historical 15/7.5 sensitivity grid, based on prior <15 psi and <7.5 psi·mL “chemical” thresholds, showed a similar pattern (Fig. 2, right panel). Under this scheme, 34 % of onset events occurred in the low-ΔP/low-W cell, 9 % in low-ΔP/high-W, 10 % in high-ΔP/low-W, and 48 % in high-ΔP/high-W. Censored events were dominated by high-dose responses: only 8 % fell in low-ΔP/low-W, whereas 80 % were in high-ΔP/high-W, with 6 % in each of the intermediate quadrants. The resulting Q1 versus Q4 odds ratio for onset versus censor was 7.08 (95 % CI 4.74–10.57; p ≈ 1.6 × 10^−12^) in the overall cohort and 4.95 (95 % CI 3.08–7.94; p ≈ 2.0 × 10^−12^) within fissured and disrupted discs.

Taken together, these quadrant analyses demonstrate that concordant pain onset in abnormal discs is primarily a low-dose phenomenon—occurring disproportionately at low static pressure and low mechanical work—whereas discs that never declare typically tolerate substantially higher pressures and work, occupying the high-ΔP/high-W quadrant.

Underlying Q1 vs Q4 counts for onset and censored events are provided in Supplementary Table S2.

### 3.5 Morphology-stratified ΔP–Work dose maps

**Fig. 3:**
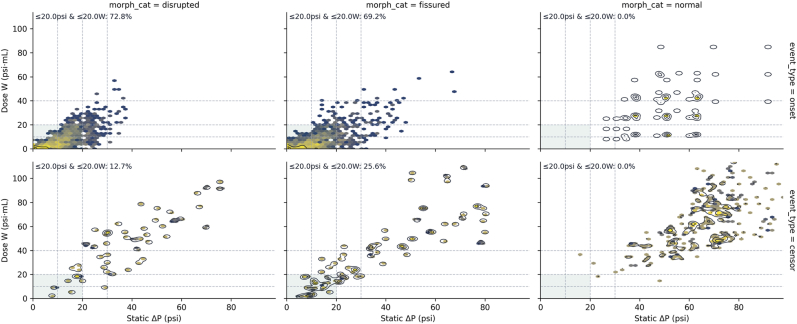
Morphology-stratified ΔP–work dose maps for onset and final-negative (censored) events. Columns represent MRI morphology (disrupted, fissured, normal controls), and rows represent event type (onset, censor). Each panel plots static pressure above opening (ΔP, x-axis) against cumulative work (W, y-axis) at the event dose, with hexbin density shading and contours indicating local point density. The shaded rectangle marks the low-dose region (ΔP ≤ 20 psi and W ≤ 20 psi·mL), and text in each panel reports the percentage of events falling within this region. In disrupted and fissured discs, the majority of onset events lie in the low-dose box, whereas only a minority of censored events do; normal control discs show no onsets in this low-dose region, and both onset (when present) and censored events occur at higher ΔP and W.

Morphology-stratified ΔP–work dose maps provided a complementary view of the separation between onset and final-negative events (Fig. 3). For disrupted and fissured discs, most onset events clustered in the low-dose region defined a priori by static ΔP ≤ 20 psi and W ≤ 20 psi·mL, whereas only a minority of censored events fell in this box. In contrast, morphologically normal control discs showed no onsets in the low-dose region, and both onset (when present) and censored doses occupied substantially higher ΔP and W values.

**Fig. 4:**
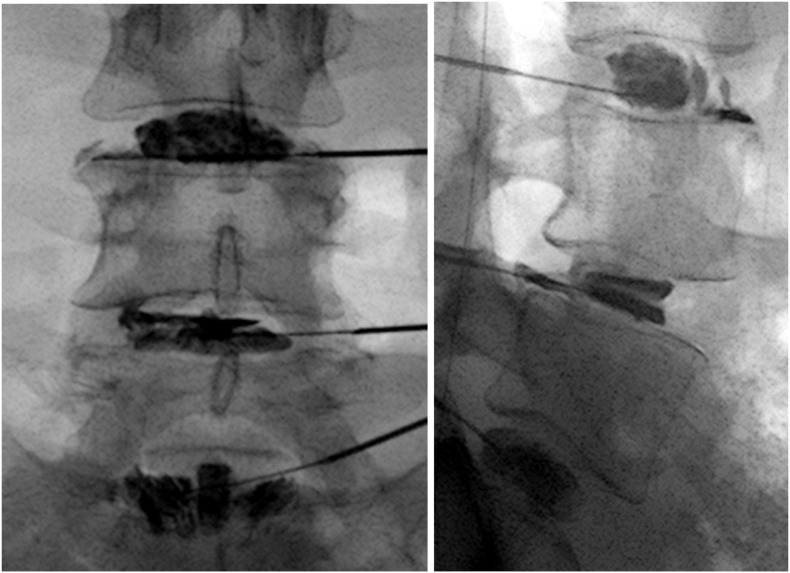
**Representative discogram illustrating relatively low-dose, high-intensity onset at L4–5 with negative adjacent levels.** AP and lateral fluoroscopic views show two-needle discography at L3–4, L4–5, and L5–S1. At L4–5, the opening step (0.2 mL, 14 → 7 psi) is followed by the first 0.5 mL increment (0.5 mL, 30 → 23 psi), which provokes familiar left-sided low back pain at 7/10 intensity as contrast flows to the outer annulus—illustrating a relatively low-dose, high-intensity onset. In contrast, at L3–4 and L5–S1, contrast remains contained within the annulus (L3–4 with a grade-3 anterior fissure, L5–S1 with a contained pattern), and no pain is provoked despite plateau pressures approaching 50 psi above opening. Clinically, this middle-aged patient had a two-year history of persistent left-sided low back pain unresponsive to extensive conservative care and medial branch neurotomy following a violent torsional injury. A recent sagittal T2-weighted MRI showed only slight loss of signal intensity at L4–5 without a discrete disc protrusion—an appearance that would lead many clinicians to dismiss the disc as a primary source of pain. Inspection of the L4–5 discogram reveals a left paracentral grade-3 fissure with contained contrast tracking parallel to the superior endplate of L5 on the lateral view, with contrast reaching the outer annulus but remaining subannular. On the AP view, a subtle tongue of contrast extends from the left outer edge of the central contrast mass toward the inferior L4 endplate, which could represent a separate, smaller annulus–endplate junctional injury from the same torsional event. Given the torsional mechanism of injury, these patterns are compatible with annulus–endplate junctional disruption and possibly a small fragment of annulus–endplate complex within the left fissure, although such a lesion typically cannot be confirmed with imaging alone. Together, these findings document how, under pressure–volume–controlled conditions, structurally abnormal but asymptomatic discs can coexist with an adjacent disc that declares abruptly at low volume and low ΔP with high-intensity, concordant pain—providing a plausible and physiologically consistent explanation for this patient's ongoing symptoms and illustrating a sensitized annular–junctional phenotype.

A small minority of final-negative (censored) events fell within the low-dose region (ΔP ≤ 20 psi and W ≤ 20 psi·mL) in these maps. Chart review of representative cases indicated that such levels typically reflected injections in which pain was present but not clearly documented as concordant in the original reports. For the purposes of this analysis, these levels were conservatively treated as censored rather than reclassified as onset positive. They should therefore be interpreted as early-stopped or ambiguous levels, rather than as true high-dose–tolerant discs.

Viewed alongside the quadrant analyses, these morphology-stratified maps reinforce the quadrant analyses by showing that, within abnormal discs, concordant pain onset arises predominantly from low-pressure, low-work stimulation, while non-declaring discs populate the higher-dose portions of the ΔP–work plane. Normal discs form a separate pattern in which even high-dose provocation rarely produces concordant pain.

### 3.6 Within-encounter paired comparisons of onset and final-negative doses

To assess whether the separation between onset and final-negative doses persisted within the same clinical and apparatus context, we examined discography encounters that contained at least one positive (onset) and at least one negative (censored) disc and had complete ΔP and W data for both event types. Among these mixed encounters (n = 151), there were 227 onset levels and 235 censored levels.

When all levels in this mixed-encounter subset were considered together, median ΔP_event was 15.9 psi for onset levels versus 32.0 psi for censored levels, and median W_event was 9.64 psi·mL versus 23.6 psi·mL, respectively. To avoid overweighting encounters with many injected discs, we also summarized doses at the encounter level, using the within-encounter median ΔP_event and W_event for onset and censored discs separately. Across encounters, the median of these encounter-level medians was 16.2 psi for onset versus 30.9 psi for censored levels, and 9.86 versus 24.4 psi·mL for W_event.

In approximately three-quarters of mixed encounters, the encounter-level median ΔP_event was lower for onset than for censored levels, and a similar proportion showed lower median W_event for onset. Despite these conservative summaries, paired Wilcoxon signed-rank tests on the encounter-level differences demonstrated highly significant separation: median ΔP_event for censored levels exceeded that for onset levels (p ≈ 1.4 × 10^−14^), and median W_event for censored levels similarly exceeded onset W_event (p ≈ 3.1 × 10^−11^).

These within-encounter findings confirm that positive discs generally declare at lower static pressure and lower mechanical work than negative discs in the same patient, under the same injector and apparatus conditions, and therefore are unlikely to be explained by inter-patient variability or systematic device bias.

## 4 Discussion

### 4.1 Overview of main findings

Using the onset and final-negative (censored) definitions and ΔP/W_event metrics described in Methods, we asked how the doses at which discs first produced definite concordant pain (“onset” steps) compared with the highest doses tolerated by discs that never declared (“final-negative” or censored steps), under a pressure–volume window of static ΔP ≤ 50 psi above opening and injected volume ≤3.5 mL. We then related these doses to disc morphology, static pressure, cumulative mechanical work, and stiffness-normalized metrics, and assessed whether the observed patterns were consistent within encounters and robust to variations in procedural conditions. In particular, all analyses were restricted to discs injected manually with a pressure-monitored Merit system, as described in the Methods.

Several consistent themes emerged. First, at the disc level, structurally abnormal discs were much more likely to declare than morphologically normal controls, reproducing the classic discography pattern in a dose-resolved framework. Fissured and disrupted discs showed high onset rates, whereas morphologically normal discs were rarely positive despite often tolerating high ΔP and work. Within abnormal morphologies, discs that declared did so at relatively modest pressure and work levels, whereas non-declaring discs in the same categories typically only reached our safety caps at substantially higher ΔP and W. Morphologically normal discs clustered at the opposite extreme, with both onset (when present) and censored doses occurring at very high ΔP and work (Table 1, Table 2).

Second, when onset occurred, it was almost always a clinically robust event. Among onset-positive levels with complete pain ratings, only a small fraction declared at ≤4/10 intensity, whereas the vast majority reached at least 5/10 and nearly three-quarters were ≥7/10 at the first qualifying step (Fig. 1A). These distributions were similar across fissured and disrupted discs and even in the small subset of positive normal discs. Companion analyses showed that onset generally arose after only a small number of additional 0.5 mL increments once the disc entered the low-pressure range, suggesting that concordant pain emerged abruptly rather than as a gradual “wind-up” at low intensities (Supplementary Fig. S1B).

Third, joint ΔP–work analyses and within-encounter comparisons confirmed that the separation between onset and censored doses was not an artifact of patient or injector differences. ΔP × work quadrant plots showed that onset events in abnormal discs were strongly enriched in the low-ΔP/low-work quadrant, whereas censored events clustered in the high-ΔP/high-work quadrant under both our protocol grid and a historical 15 psi/7.5 psi·mL sensitivity grid (Fig. 2). Odds ratios for onset versus censor in the low-versus high-dose quadrants were large and remained highly significant when restricted to fissured and disrupted discs. Encounter-level paired analyses yielded the same pattern: in mixed encounters containing both positive and negative discs, within-encounter median ΔP and work at onset were consistently lower than those at the final-negative step, with highly significant differences on paired nonparametric testing. Stiffness-normalized checks (K_e_q) showed similar median values for onset and censored events within morphology groups, indicating that the observed separation reflects differences in the dose at which pain appears, not merely differences in disc “softness.”

Overall, these findings support a simple but important conclusion: under pressure–volume–controlled conditions, a subset of structurally abnormal discs reliably declares with high-intensity concordant pain at relatively low static pressure above opening and low mechanical work, while other discs—whether morphologically normal or dominated by alternative generators—tolerate much higher doses without reproducing the presenting symptoms. In the sections that follow, we interpret these patterns in the context of disc injection mechanics, disc and endplate innervation, chemical sensitization, and imaging correlates, and we propose an annular versus vertebrogenic framework for understanding how pressure-controlled discography can contribute to the evaluation of anterior column pain.

Earlier pressure-controlled investigations—including our facility's initial IPSIS discometry series [10,12] and the subsequent dynamic manometry analyses by O'Neill, and colleagues [17,22]—showed that discs differ systematically in their pressure–response behavior and helped establish several of the physiologic principles that underpin contemporary interpretations of discogenic pain. The early work using static pressure–volume measurements in both side-lying and sitting positions demonstrated that concordant pain in fissured or degenerated discs typically emerged at low rises above opening pressure (ΔP), often near 15 psi, whereas other discs required higher ΔP (approximately 30 psi) to respond, with few levels needing more than 50 psi above opening; these observations were formalized into a pragmatic 0–4 pressure-based grading system that distinguished high-intensity low-ΔP (“chemical”) responses from high-intensity higher-ΔP (“mechanical”) responses and related these phenotypes to surgical outcomes [13]. Building on that foundation, the present analysis treats disc provocation as a fully dose-resolved experiment, using static plateau ΔP, cumulative mechanical work (W), stiffness-normalized metrics, and direct comparison of onset and final-negative (censored) doses within and across MRI morphologies to assess whether low-dose annular responses reliably separate from the tolerance profiles of non-declaring levels and whether these patterns remain robust across morphology, apparatus conditions, and disc stiffness.

The present analysis can be viewed as a larger, more rigorously quantified extension of those early prospective pressure-controlled series, initially reported in the Interventional Spinal Injection Society scientific bulletins, which first introduced the 0–4 pressure-based discogram classification and the 15–30–50 psi thresholds that are examined here in more detail.

Collectively, these findings are consistent with our prespecified hypotheses. In MRI-abnormal discs, concordant pain onset occurred at substantially lower ΔP and W than the final-negative doses tolerated by non-declaring discs, and this separation persisted within morphology strata, within encounters, and after stiffness normalization. The patterns were similar across injector conditions, supporting the view that the dose–response behavior reflects disc and segment physiology rather than apparatus artifacts.

We recognize that discography remains controversial, particularly with respect to false-positive responses in morphologically abnormal but clinically silent discs and concerns about iatrogenic disc injury [[23], [24], [25]]. The present retrospective analysis does not address long-term structural consequences and was not designed to estimate false-positive or false-negative rates in a population sense. Instead, our goal was to examine how pressure–volume behavior differs between discs that do and do not reproduce the patient's pain under strict criteria, and to place those patterns into a physiologic framework that is compatible with current mechanistic and imaging data. The findings should therefore be interpreted as construct validation for annular and vertebrogenic pain models rather than as a global referendum on discography. Nevertheless, subsequent psychometric work and a systematic review of asymptomatic discography suggest that, when modern pressure limits and interpretive criteria are applied, false-positive rates remain low and are not substantially driven by chronic pain status or psychological distress [26,27]. Given the long-standing controversy around discography and the increasingly recognized overlap between annular and vertebrogenic mechanisms, we have therefore organized the Discussion into focused subsections that address the mechanical, innervation, inflammatory, natural history, and imaging data needed to interpret these dose–response patterns in a physiologically coherent way.

### 4.2 Mechanical and nociceptive effects of disc injection

#### 4.2.1 Mechanical behavior of injected, degenerated discs

Cadaveric injection studies have shown that intradiscal contrast does not simply “pressurize” the disc as a uniform hydraulic chamber. Instead, injected fluid initially displaces the matrix and forms discrete pools whose size and location depend on the stage of degeneration and the presence of fissures. Adams and colleagues injected dye–contrast mixtures into cadaveric lumbar discs and then sectioned them, demonstrating that in non-degenerated “cottonball” discs, fluid remains confined within a soft, amorphous nucleus and forms a continuous system of small pools, whereas in more degenerated discs it tracks along clefts, inner annular fissures, and radial tears to form larger pools in the annulus and beneath the endplates [28]. These distribution patterns corresponded closely to classic discogram appearances (cottonball, lobular, irregular, fissured, ruptured) and provided direct anatomic validation that discograms reflect the internal architecture of the disc rather than arbitrary contrast spread. Adams et al. also emphasized that the disc makes room for injected fluid primarily by bulging of the endplates and annulus, and that injection pressures depend on the resistance of these structures to deformation: young, non-degenerated discs accepted smaller volumes and required higher injection pressures, whereas older, degenerated discs accepted larger volumes at lower pressures.

In vivo and cadaveric stress-profilometry work by Adams and McNally provides complementary physiologic evidence that discogenic pain is associated with abnormal, focal annular loading rather than simply uniformly elevated intradiscal pressure. Cadaveric measurements showed that degeneration produces markedly abnormal internal load distributions, with focal increases in annular stress and a loss of normal hydrostatic support within the nucleus [28,29]. McNally and colleagues then extended these observations clinically by using miniature stress transducers passed through a cannula into the discs of symptomatic patients during discography to map stress at multiple positions within the nucleus and annulus [30]. In these in vivo measurements, painful discs showed pronounced stress concentrations within the posterolateral annulus and more heterogeneous nuclear stress distributions compared with non-painful discs, consistent with the cadaveric findings of abnormal annular loading and nuclear depressurization [29]. Stress peaks appeared in the annulus at similar magnitudes across vertical positions, rather than being confined to regions immediately adjacent to the endplates—a pattern more consistent with shear and focal loading in the annular wall and its attachments at the annulus–endplate junction than with simple endplate indentation or uniformly increased compression [30]. Given that the transducers were advanced along a track through the mid-disc and then withdrawn, some of these focal peaks likely reflect transitions between solid annular tissue, fissure cavities, and junctional regions along that path, further emphasizing the heterogeneous mechanical environment of the degenerated annulus–endplate complex.

Viewed together, the anatomic injection studies of Adams and the stress-profilometry work of McNally support a common mechanical interpretation: degeneration converts the disc from a nearly hydrostatic, nucleus-dominated structure into one in which loads are carried by a fissured, nociceptor-bearing annulus, with injected fluid preferentially tracking along fissures and clefts. In such discs, small changes in injected volume or segment position can cause abrupt local shifts of load from fluid-supported regions to solid lamellae and back again, creating sharp local increases in shear and tensile strain at fissure margins—particularly where nuclear material has migrated into the tear or where fissures and rim (avulsion) lesions involve the annulus–endplate junction.

Our pressure–volume data extend this concept at the clinical scale. In structurally abnormal discs, concordant pain onset usually occurs at low global ΔP and low cumulative work, yet at high pain intensity, whereas discs that never declare tolerate substantially higher pressures and work. This pattern is exactly what would be expected if pain reflects the disc's transition from hydrostatic, nucleus-dominated behavior to a shear-dominant state, in which injected fluid and physiologic loads act on a disrupted, innervated annulus and its attachments at the annulus–endplate junction. In contrast, discs that retain a largely hydrostatic nucleus and intact annulus can accommodate injected fluid with relatively uniform deformation and few focal stress peaks; such discs in our series generally tolerated higher ΔP and work without concordant pain.

#### 4.2.2 Sensory and sympathetic innervation of disc and endplate

Chemical sensitivity presupposes that nociceptive nerve fibers are present in the tissues being stimulated [6]. In the healthy spine, sensory innervation of the lumbar disc is largely confined to the outer annulus and posterior longitudinal ligament, supplied by the sinuvertebral nerve and branches from the gray rami communicantes [6,7]. The sinuvertebral nerve is a recurrent mixed nerve that arises from the ventral ramus and gray ramus, re-enters the spinal canal, and provides predominantly small myelinated and unmyelinated sensory and sympathetic fibers to the posterior annulus, posterior longitudinal ligament, periosteum, and dura. The inner annulus and nucleus pulposus are essentially aneural in non-degenerated discs, but with degeneration nociceptive fibers and blood vessels can grow centripetally along fissures and clefts into the inner annulus and even the nuclear region [6,7].

Freemont and colleagues demonstrated this ingrowth convincingly in disc biopsy material from patients undergoing fusion for chronic back pain. In their Lancet series, deep nerve ingrowth into the inner third of the annulus and into the nucleus pulposus, with fibers expressing substance P and the growth-associated protein GAP43, was significantly more common in discography-defined pain levels than in adjacent non-pain levels or normal controls [31]. These findings show that normally aneural regions of the disc can become innervated by actively growing nociceptive fibers, and that such ingrowth is strongly associated with clinically painful discs.

The vertebral endplates and adjacent vertebral body are innervated through a related but anatomically distinct pathway. An intraosseous basivertebral nerve courses within the vertebral body and through the basivertebral foramen to supply the superior and inferior endplates and adjacent marrow. Vertebrogenic pain is thought to arise when endplate microdamage and marrow changes activate these basivertebral fibers, in contrast to annular pain transmitted primarily via sinuvertebral and gray-ramus afferents. Both systems have a substantial sympathetic component and can be exposed to inflammatory mediators originating in the degenerating disc. For purposes of exposition, we refer to “annular” (sinuvertebral/gray-ramus) and “vertebrogenic” (basivertebral/endplate) nociception as if they were separable. Anatomically, these systems supply closely apposed territories at the outer annulus, the annulus–endplate junction, and adjacent vertebral marrow, and histologic studies demonstrate nociceptors in each of these regions [6,8,9,31]. Direct anatomic tracing of continuous sinuvertebral–basivertebral fibers is limited, but their spatial proximity and shared exposure to disc- and marrow-derived inflammatory mediators make it likely that both systems can sample the same sensitized tissue volumes. Taken together, anatomic and histologic studies indicate that for a disc to be chemically sensitive in the way our low-dose onset data suggest, nociceptive fibers must first invade or lie adjacent to the disrupted annulus and/or endplate interfaces; structural change alone is not sufficient unless it occurs in regions that are actually innervated. Within this shared disc–endplate–vertebral nociceptive zone, pressure–volume–controlled discography is best viewed as identifying discs in which low-dose onset is most consistent with annular and annulus–endplate junctional sensitization, rather than proving that a single named nerve pathway is solely responsible.

#### 4.2.3 Chemical drivers of nociceptor sensitization

Once nociceptive fibers are present in the annulus or endplate region, the local chemical milieu becomes a key determinant of pain sensitivity. Disc degeneration is characterized by increased production of pro-inflammatory cytokines (TNF-α, IL-1β, IL-6), prostaglandins, matrix-degrading enzymes (MMPs, cathepsins, ADAMTS), and neurotrophins such as NGF and BDNF, all of which contribute to inflammation, matrix breakdown, and neuronal sensitization [[32], [33], [34], [35], [36], [37]]. These mediators promote ingrowth of blood vessels and nerve fibers into fissured annulus and adjacent endplate marrow and lower the activation threshold of existing nociceptors.

In disc tissue specifically, NGF expression has been shown to localize to microvascular endothelial cells and ingrowing nociceptive fibers in painful discs, whereas non-painful and control discs show little or no NGF expression [33]. Toll-like receptors are expressed and regulatable in human disc cells, with TLR2 and TLR4 activation modulating inflammatory signaling [38]. Krock and colleagues further demonstrated that TLR2 activation in human NP and AF cells from non-degenerate donor discs directly up-regulates NGF (and BDNF) via an NF-κB–dependent pathway, independent of secondary IL-1 signaling, providing a mechanistic link between matrix-derived “alarmins,” inflammatory signaling, and NGF production in the disc [39]. Taken together, these findings support a model in which matrix breakdown and low-grade inflammation generate TLR2/NF-κB–driven NGF expression within annulus and endplate tissues, promoting nerve ingrowth and lowering nociceptor activation thresholds. In such chemically sensitized discs, even modest increases in intradiscal pressure during pressure–volume–controlled injection are sufficient to provoke high-intensity, concordant pain at low ΔP and work—precisely the pattern observed in our low-dose discometry phenotype.

At the same time, mechanical micro-instability at the fissure—repetitive shear and tensile strain at the margins of a radial or circumferential tear—interacts with this inflammatory milieu. Cyclic loading of a disrupted disc can repeatedly deform sensitized fibers embedded in granulation tissue or vascularized fissures, generating ongoing nociceptive input even at modest global pressure levels [[40], [41], [42], [43], [44], [45]]. This combination of chemical and mechanical stimuli provides a plausible substrate for the low-pressure, high-intensity onset events observed in our cohort: relatively small increases in intradiscal pressure during pressure–volume–controlled injection may be sufficient to trigger firing in already sensitized nociceptors in the annulus and at the annulus–endplate junction, even though the overall ΔP and work remain well below the levels tolerated by non-sensitized discs.

#### 4.2.4 Resolution versus chronic symptoms: why some discs stay painful

Clinically, many annular fissures are transiently symptomatic or never become painful, whereas a subset progresses to recurrent or chronic discogenic pain. This heterogeneous natural history likely reflects differences in how peripheral and central sensitization evolve. In some discs, inflammatory activity is short-lived or effectively controlled by host responses; nociceptor density and neuropeptide expression regress, and the fissure heals structurally or becomes mechanically “quiet” [9,44,45]. In others, persistent local inflammation, repeated mechanical provocation, or episodes of neuritis maintain ongoing nociceptor activity and peripheral neuroinflammation [44,46,47]. Experimental and clinical work in spinal pain models indicates that such sustained peripheral input can sensitize dorsal root ganglion neurons and dorsal horn circuits, lowering thresholds and amplifying responses to subsequent stimuli and thereby promoting a transition from acute, load-linked pain to chronic or intermittently recrudescent pain [9,48,49]. In that sensitized state, relatively modest changes in disc loading—or even non-disc inputs—can reactivate pain in a previously symptomatic motion segment. For our study, this natural history implies that a disc demonstrating low-dose, high-intensity onset at the time of discography is likely to be in a sensitized state; whether that state persists, remits, or evolves over time is not addressed by the present retrospective dataset and remains an important limitation when extrapolating from provocation responses to long-term prognosis.

#### 4.2.5 Shared inflammatory milieu of disc and endplate

Disc and vertebral endplate tissues do not exist in isolation. Degeneration of the disc and microdamage to the endplate are strongly coupled processes: fissures and nuclear depressurization alter load transfer to the endplate, while endplate microfractures and Modic changes reflect bidirectional biochemical exchange between the disc and vertebral marrow. Histologic studies of degenerative motion segments have documented increased vascularity and sensory nerve proliferation not only in annular fissures but also in endplate cartilage and adjacent vertebral bone, with expression of substance P, CGRP, and related neuropeptides [50]. Mapping of human lumbar vertebrae and endplates has shown dense PGP 9.5–positive innervation not only in the outer annulus but also in endplate cartilage and adjacent vertebral marrow, particularly in association with endplate defects and Modic-like lesions [8]. The NGF-expressing microvessels described by Freemont and colleagues, which enter the disc through the endplates and are accompanied by TrkA-positive, GAP43-positive nociceptive fibers, illustrate this shared environment particularly well, linking vertebral marrow–derived neovascularization, discal neurotrophin signaling, and deep disc innervation within a single motion segment [33]. This integrated model is further supported by contemporary reviews synthesizing animal and human data showing that sensory nerve ingrowth, cytokine upregulation (TNF-α, IL-1β, IL-6, NGF), and segmental hypermobility together form the core pathophysiologic triad of discogenic pain [9].

From the standpoint of pressure-controlled discography, this mixed environment implies that injected contrast and pressure may stimulate both annular and endplate-related nociceptors, depending on the degree of fissuring, endplate permeability, and nerve ingrowth [8,9]. If nerves have not penetrated annular tears—either because fissures are shallow or because ingrowth has not occurred—the disc may remain asymptomatic despite marked structural degeneration and Modic change [51,52]. Conversely, when nociceptors are present in both fissured annulus and inflamed endplate, chemical and mechanical stimuli will likely co-activate annular and vertebrogenic pathways, producing a blended pain experience [8,9]. Collectively, anatomic and histologic studies demonstrate that sinuvertebral fibers supplying the annulus and basivertebral branches supplying the endplate penetrate adjacent and partially overlapping regions of the annular–endplate interface, and that nerve ingrowth and inflammatory mediators concentrate in these regions [6,8,9,31,50]. Inflammatory mediators and nerve ingrowth can further blur these boundaries, creating a shared nociceptive zone in which either system may sample the same sensitized tissue volume depending on local loading and biochemical conditions.

Animal models summarized by Lotz and Ulrich reinforce this view: loss of nuclear pressurization appears to be the initiating event in disc degeneration, but painful, or pathologic, degeneration is characterized by ineffective healing of the peripheral tissues at the endplate and outer annulus—precisely the sites where vascular access, inflammation, neoinnervation, and nociceptor sensitization can occur [53].

Taken together, this anatomic and biochemical evidence implies that disc and endplate lesions share a common nociceptive substrate, but the specific pressure–volume patterns observed in our cohort—low-dose, high-intensity onset in fissured and disrupted discs, high-dose tolerance in non-declaring discs, and comparable stiffness across onset and censor events—are most consistent with a predominantly annular–junctional nociceptive subset at the doses used here, operating within a broader, mixed disc–vertebral pathology.

#### 4.2.6 Biologic basis for low-dose, contact-dependent onset

Experimental and histologic studies provide a plausible biologic substrate for the low-dose, high-intensity onset pattern observed in this cohort. Reviews synthesizing human and animal data show that disc degeneration promotes sensory nerve ingrowth from the outer annulus toward the inner annulus and endplate, upregulation of cytokines such as TNF-α, IL-1β, IL-6, and NGF, and hypermobility of the motion segment [9]. Together, these changes lower nociceptor activation thresholds and create a chemically and mechanically sensitized disc–endplate unit rather than a purely mechanical “shock absorber” [6,31]. Within such a sensitized environment, even modest increases in global pressure or shear would be expected to provoke pain once local loads at nerve-bearing regions cross a critical level, whereas structurally similar but less inflamed discs may remain tolerant at the same dose [54].

More recent high-resolution mapping of human discs supports a specifically contact-dependent model of nociceptor activation. Lama and colleagues used confocal microscopy and immunofluorescence to show that in surgically removed degenerated and herniated discs, sensory nerves and blood vessels were confined to proteoglycan-depleted, physically disrupted annulus tissue, typically within 0.14–0.25 mm of fissure surfaces and never within intact annular lamellae or normal nucleus [54]. Nerves tracked along fissure walls and matrix defects and were absent from regions without structural disruption or focal GAG loss. These findings indicate that nociceptors reside precisely at the fissure margins and annular–endplate interface—locations that injected contrast or fluid will encounter early during discography, well before global ΔP and work become high.

Our discometry results are consistent with this histologic framework. In fissured and disrupted discs, concordant pain onset occurred at low static ΔP and low cumulative work, yet with high intensity, whereas many structurally abnormal discs tolerated much higher doses without declaring. Histologic studies do not prove that our specific low-dose onset events necessarily arise from the same nerves observed in surgical specimens, nor do they allow us to assign pain uniquely to sinuvertebral versus basivertebral pathways. However, they support the interpretation that low-dose onset reflects threshold crossing at sensitized nerve endings located on fissure and junctional surfaces, rather than diffuse pressurization of a homogeneous disc, and they make it biologically plausible that therapeutic agents will be most effective when delivered directly to these nociceptive zones. Within this biologic context, the abrupt onset of intense concordant pain at relatively low global ΔP and work is most naturally interpreted as a local threshold being exceeded at sensitized fissure and junctional sites rather than as gradual wind-up, an interpretation that is reflected in the shape of the onset distributions and stepwise pressure–volume behavior in our cohort.

#### 4.2.7 Threshold versus wind-up: conditions under which contrast injection provokes pain

The intensity and timing of concordant pain onset in this cohort (Section 3.3; Supplementary Fig. S1) argue for a threshold-based response in sensitized discs rather than a gradual central “wind-up” effect. Under our protocol, injection was slow and stepwise, with static plateaus at each 0.5 mL increment and ample time between steps. Within this framework, onset-positive discs generally remained minimally symptomatic or silent through the early steps and then showed an abrupt jump to clearly positive pain at a particular step—most often in the 6–8/10 range—rather than drifting upward over several low-intensity increments. Only a small minority of discs declared at low intensity, and the majority reached high-intensity concordant pain at the first qualifying onset step. This pattern is difficult to reconcile with a simple central wind-up mechanism driven by repeated high-frequency nociceptor firing during the test itself, but it is exactly what would be expected if local mechanical and chemical conditions at a fissure or annulus–endplate interface cross a peripheral threshold at a specific pressure–volume combination, consistent with the practical volume-staging behavior described in Section 4.6.

Within a structurally disrupted disc that contains sensitized nociceptors, injected contrast and fluid provoke pain not primarily because of the chemical properties of the injectate but to the localized mechanical effects of pressurization [17,40,43]. Local pain-mapping studies similarly show that concordant pain occurs only when contrast reaches sensitized neural tissue, whereas identical injections on the contralateral side remain silent [55]. Together, these observations support the broader concept that only targeted delivery to the precise nociceptive site—rather than diffuse injection—is capable of activating (or, therapeutically, quieting) sensitized neural elements.

As Adams and colleagues showed, fluid tracks along fissures and clefts and forms discrete pools in the annulus and beneath the endplates rather than simply pressurizing a homogeneous nucleus [28]. Even modest global pressure increases can therefore cause abrupt local changes in shear and tensile strain at the margins of a fissure, particularly when nuclear depressurization has shifted load onto a disrupted, nerve-bearing annulus. In that setting, a small additional volume at a particular step can be enough to tip local stress at sensitized nerve endings past a critical threshold, producing a sudden, high-intensity pain response despite relatively low overall ΔP and work.

Conversely, in discs that lack deep nociceptor ingrowth—or in which annular and endplate nociceptors are not chemically sensitized—the same pressure–volume profile produces little or no pain. Contrast spreads within the matrix and along fissures, but the local deformation of innervated tissue remains below the threshold needed to generate a strong nociceptive signal. The fact that morphologically normal discs in our series often tolerated high ΔP and work without concordant pain, and that even many fissured or disrupted discs remained negative under the protocol caps, underscores this point: injected contrast is not inherently noxious. It becomes painful when it is used as a controlled way to reproduce the shear and micro-instability experienced by sensitized annular or endplate nociceptors at their threshold for activation.

Seen this way, the high-intensity onset distributions in our data are not incidental; they are part of the core construct that pressure-controlled discography is probing. The test does not gradually “wind up” pain in otherwise quiescent discs. Rather, it identifies discs in which structural disruption, nerve ingrowth, and chemical sensitization have created a state where modest, well-bounded changes in pressure and volume are sufficient to trigger a threshold event that closely mimics the patient's presenting pain.

### 4.3 Annular versus vertebrogenic subsets of anterior column pain

The mechanical picture emerging from prior injection and stress-profilometry studies, together with the present pressure–volume findings, is most easily understood if anterior column pain is conceptualized as comprising at least two recognizable phenotypes within a functionally overlapping annular–endplate–vertebral nociceptive field: annular-dominant and vertebrogenic-dominant pain. Both arise within the same motion segment and often coexist, but they differ in their predominant tissue source, loading conditions, and response to pressure-controlled provocation. In this framework, annular and vertebrogenic labels describe functional dominance rather than strict anatomic isolation of sinuvertebral versus basivertebral fibers.

Annular tears and fissures are common features of disc degeneration, yet most structurally abnormal discs are not painful. Pain appears to require not only disruption of the annular architecture but also the presence of nociceptive fibers within or adjacent to the fissure and sufficient chemical and mechanical stimuli to sensitize those fibers [56]. Experimental and histologic work has shown that nerve ingrowth into the outer annulus and even into the nuclear region can occur along fissure tracks and that these ingrown fibers express nociceptive neuropeptides and inflammatory mediators. Earlier CT/discography work using pressure-controlled provocation supports this annular nociception model: in a manometric series, symptomatic discs were strongly associated with CT-evident disruption of the outer annulus, whereas lower-grade disruptions were rarely painful [16]. The present pressure–volume analysis extends that work by demonstrating that, within structurally abnormal discs, concordant pain onset typically occurs at low global ΔP and low cumulative work yet at high intensity, reinforcing the view that outer annular disruption in a nerve-bearing zone is a key determinant of discogenic pain. As discussed in the mechanics section, in vivo stress-profilometry studies by McNally and colleagues provide complementary support: painful discs showed abnormal loading of the posterolateral annulus with focal stress concentrations and a depressurized nucleus, whereas painless discs retained a more uniform, nucleus-centered stress pattern, and it was this abnormal annular loading—not peak stress magnitude alone—that predicted pain on discography [30].

Vertebrogenic pain, in contrast, is thought to arise primarily from load-dependent mechanisms involving endplate microdamage, marrow changes, and altered stress transfer into the vertebral body [57,58]. Basivertebral nerve fibers within the vertebral body and endplate are believed to mediate this pain, which may be more prominent under repetitive or sustained mechanical loading and less readily provoked by modest intradiscal pressurization. An early non–pressure-controlled series by Hsu and colleagues reported that lumbar discs with fluoroscopic endplate disruptions and contrast leakage into the vertebral body showed a higher frequency of moderate to severe concordant back pain than discs without such disruptions, leading the authors to suggest that endplate injury “may be related to painful segments” [59]. Modic changes and other vertebral signal alterations on MRI are now frequently cited as markers of vertebrogenic pain, yet they almost always occur in the setting of coexisting disc degeneration and annular disruption, so a substantial fraction of such segments likely have both annular and vertebrogenic contributions rather than a single isolated generator [60].

The patterns observed in this study fit naturally into this two-subset framework. In fissured and disrupted discs, concordant pain onset clustered at the lower end of our pressure and work range and, when it occurred, the pain was usually high intensity, consistent with activation of sensitized annular nociceptors under shear-dominant loading conditions. Discs that never declared—often in the same encounter—tolerated much higher ΔP and W without reproducing the patient's symptoms, whereas morphologically normal discs sat at the opposite extreme, rarely producing concordant pain and doing so only at high doses. Stiffness-normalized checks (K_eq) showed that onset and censor events had similar median stiffness within morphology groups, indicating that the separation is not simply due to “soft” discs being more likely to declare. Instead, the data are more consistent with the idea that a subset of structurally abnormal discs has become chemically and mechanically sensitized at the annular level and responds to relatively modest global pressure–volume loading with high-intensity pain, whereas other discs—whether truly normal or dominated by vertebrogenic mechanisms—remain silent or require much higher loads to provoke symptoms.

This annular–vertebrogenic distinction does not imply mutually exclusive categories, nor does it diminish the potential importance of vertebral endplate pathology. Rather, it provides a useful conceptual scaffold for interpreting pressure-controlled discography within an overlapping SVN–BVN nociceptive landscape. Low-dose, high-intensity concordant pain in a structurally abnormal disc is difficult to explain without invoking sensitized annular and annulus–endplate junctional nociceptors as major contributors, whereas high-dose tolerance in the same spine suggests alternative or coexisting generators such as vertebrogenic pain, facet or sacroiliac sources, or clinically silent degeneration. Our intent is not to reassign most discogenic pain to any single mechanism, but to use dose–response behavior to indicate which territory is likely dominant at the time of testing.

### 4.4 Relation to prior discography and discomanometry literature

These observations are broadly consistent with earlier pressure-controlled discography literature that reported concordant pain at relatively low pressures in structurally abnormal discs and high-pressure tolerance in morphologically normal controls [14,15,22]. A historical “chemical disc” pressure threshold of ΔP <15 psi, together with our conservative choice of a low-work band (W < 7.5 psi·mL), anticipates the idea that some discs become symptomatic at modest loading, whereas others require much higher pressure and volume to provoke pain, if they respond at all [10]. Our data place these concepts into a full pressure–volume–resolved framework, showing that onset doses in fissured and disrupted discs cluster at low ΔP and W, while censor doses often exceed both the historical chemical thresholds and our protocol caps without reproducing the patient's symptoms (Table 1, Table 2; Fig. 2-).

Prior pressure-controlled CT/discography work using the same ≤50 psi and low-volume criteria has linked structural disruption of the outer annulus with symptomatic discs under controlled loading, supporting an annular nociception model [16]. Independent series using manometric discography in surgical candidates have reported compatible pressure–response relationships. Shin et al. [61] used a pressure-controlled system in 21 patients (51 discs) and found that disc elastance (ΔP/ΔV) was significantly lower in degenerated discs and that pain responses correlated with intradiscal pressure rather than injected volume; at a given morphologic grade, positive discs showed pain at lower pressures and higher intensity (VAS ≥6), whereas most negative discs remained low-intensity even at 50 psi. These findings reinforce the notion that it is the combination of structural degeneration and pressure-dependent loading, rather than volume alone, that defines a painful disc.

Device comparisons also support the robustness of pressure-controlled criteria. Derby et al. (2011) compared automated versus manual syringe-pump manometry in 510 discs and found equivalent overall and low-pressure positive discogram rates (32.1 % vs 32.6 % overall; 16.0 % vs 15.0 % low-pressure positive), despite lower recorded onset pressures and higher injected volumes with automated injection [62]. This suggests that, when strict pressure and pain criteria are applied, device choice does not materially alter diagnostic yield, although automated systems may provide more consistent pressure–volume stimulation. Quantitative discomanometry in percutaneous disc treatments has likewise suggested that moderate pressure and volume ranges are clinically meaningful: Filippiadis et al. [63] reported greater pain reduction when maximum intradiscal pressures remained around 60–65 psi and injected volumes were modest, implying that intradiscal pressure profiles carry prognostic as well as diagnostic information.

Several MRI-based studies have reached similar conclusions about the structural correlates of painful discs: desiccation or moderate loss of nuclear signal is more predictive of a painful disc than isolated high-intensity zones (HIZ) or small protrusions, and many HIZ-positive discs are painless on pressure-controlled discography. O'Neill et al. [64] highlighted disc desiccation as the strongest MRI predictor of positive discography, and more recent work by Jain et al. (2021) [65] found disc desiccation (Pfirrmann ≥3) to be the only MRI feature that independently predicted a painful disc on pressure-controlled provocation, whereas HIZ and protrusion/extrusion did not reach significance.

By incorporating stepwise ΔP and work, stiffness-normalized metrics, ΔP × work quadrants, and within-encounter analyses, the present study extends prior work from largely descriptive pressure–volume observations to a more quantitative characterization of annular nociception. The convergence between our findings and earlier “chemical disc” thresholds [10,13,22]; stress-profilometry studies [29,40]; outcome-based discomanometry [63]; MRI-based predictors of painful discs [22,65]; CT/discography correlations with outer annular disruption [16]; and device-comparison work in pressure-controlled discography [61,62] strengthens the argument that pressure-controlled discography, when performed carefully, is measuring a physiologically meaningful construct rather than arbitrary operator behavior.

### 4.5 Imaging, modic changes, and the limits of structural surrogates

Contemporary imaging has transformed how we visualize disc and vertebral pathology, but it does not by itself distinguish annular from vertebrogenic pain generators. MRI signal changes around the disc—whether interpreted as “inflammation,” edema, or Modic changes—are common in degenerative spines and are not inherently specific for pain. These signals only translate into annular or nuclear pain when nociceptors are present and sensitized in the degenerated tissue. Prospective MRI–discography comparisons also underscore the limitations of MRI for annular pathology. Osti and Fraser (1992) found that although all MRI-abnormal discs had abnormal discograms, 18 of 60 discs with normal MRI signals showed abnormal discograms, and they concluded that discography was more accurate than MRI for detecting annular tears and that a normal MRI does not exclude clinically relevant peripheral disc pathology [66].

Modic changes, in particular, tend to be relatively late manifestations of degeneration and are typically found in segments that already show disc height loss and annular fissuring rather than in structurally intact discs [51,67,68]. A substantial fraction of such segments likely have both annular and vertebrogenic contributions rather than a single isolated source. This dual contribution is consistent with early fluoroscopic observations that endplate disruptions with contrast leakage into the vertebral body were more often associated with concordant back pain than intact endplates in a non–pressure-controlled manual series [59]. Modic lesions and endplate marrow changes provide direct evidence that structural damage at the disc–endplate junction can generate pain through vertebrogenic pathways: Dudli and colleagues proposed a disease model in which disc and endplate damage, together with a persistent inflammatory stimulus, create predisposing conditions for vertebral bone marrow lesions visible as Modic changes [69], and Lotz and colleagues have highlighted that endplates must balance mechanical strength and porosity, making them vulnerable to microdamage and neoinnervation that are susceptible to both chemical sensitization and mechanical stimulation [70]. At the same time, subtle endplate defects and neoinnervated regions are often poorly visualized on standard MRI sequences, contributing to under-recognition of vertebrogenic pain in routine imaging.

Within this context, our pressure–volume findings can be viewed as complementary rather than competing with imaging-based assessments. In our cohort, discs showing low-dose, high-intensity onset most often behaved like segments with a sensitized annulus or annulus–endplate junction, whereas high-dose tolerance—particularly in the presence of Modic changes or suspected endplate defects—was more compatible with vertebrogenic or non-discogenic sources that may require sustained compressive loading, endplate bending stress, or transient peak forces not reproduced by pressure-controlled discography. At the same time, the convergence of discal and vertebral inflammation means that in some highly sensitized Modic type 1 segments, endplate nociceptors could plausibly respond at relatively low global ΔP and W, so that some portion of the low-dose onset events in our cohort may represent vertebrogenic or mixed disc–endplate nociception rather than purely annular pain. Because the present analysis under represents Modic discs and was not powered to stratify onset and censor thresholds by Modic status, we cannot reliably distinguish these patterns in the current dataset. Future work linking pressure–volume signatures, including dynamic pressure behavior, to detailed endplate imaging and Modic phenotypes will be required to determine when low-dose responses primarily reflect annular sensitization, when they reflect vertebrogenic mechanisms, and when both contribute meaningfully to the patient's pain.

### 4.6 Clinical implications, strengths, limitations, and future directions

Clinically, these findings support viewing pressure–controlled discography as a physiologic probe of annular-dominant nociception rather than as a simple binary test of “discogenic pain.” In a structurally abnormal disc, low-dose, high-intensity concordant pain is difficult to reconcile without invoking sensitized nociceptors located in the annulus and annular–endplate junction, the regions most likely to become sites of nerve ingrowth and chemical sensitization in degeneration. Conversely, high-dose tolerance in the same spine suggests that the patient's symptoms may arise from vertebrogenic sources, facet or sacroiliac mechanisms, or clinically silent degeneration rather than from that disc. In this sense, the data argue not for reinstating discography as a universal surgical gatekeeper, but for its selective use in complex anterior column pain presentations where multilevel degeneration, mixed disc–vertebral changes, or nondiagnostic responses to other interventions obscure the dominant generator. When treatment decisions hinge on whether a particular disc is truly nociceptive, pressure–controlled discography remains the only direct method to test for the presence of sensitized nociceptors within the disc–endplate complex—information that cannot be reliably inferred from MRI morphology, Modic changes, or lack of response to facetogenic or radicular blocks.

Importantly, the limited ability of a positive discogram to consistently predict good outcomes from fusion or other structural interventions should not be taken as evidence that the disc is not nociceptive. This distinction between identifying a painful motion segment and choosing an effective treatment is often overlooked. Even when a disc is convincingly shown to harbor sensitized nociceptors, fusion, stabilization, or intradiscal procedures may fail for reasons unrelated to the diagnosis—such as technique, patient selection, coexisting pain generators, or the natural history of discogenic pain, which can fluctuate or remit independently of the intervention. Thus, treatment failure does not invalidate the diagnostic information provided by pressure–volume–controlled discography; rather, it underscores that diagnosis and treatment efficacy are distinct questions, each requiring separate evaluation.

An additional practical implication arises from the onset step–index data. In our protocol, an opening step of approximately 0.2 mL was followed by 0.5 mL increments, each with a static plateau and patient questioning between steps. In the secondary subset of 153 onset-positive levels with complete step-index data, roughly three-quarters declared by the third 0.5 mL step (≈1.7 mL total volume), and nearly all declared between ≈1.7 and 2.2 mL (Supplementary Fig. S1). Fissured discs, in particular, showed about 80 % of onsets by step 3. This supports the idea that, in most structurally abnormal discs with sensitized annular nociception, concordant pain onset can be detected within a total injected volume on the order of 2 mL when the injection is staged in small increments. Even when formal discography is not performed, interventionalists who are planning intradiscal biologic injections may be able to obtain useful information by adopting a similar incremental approach during therapeutic disc injection—e.g., delivering 0.5 mL boluses over ≈2 s with brief pauses to ask whether the patient feels their usual pain. When such low-dose onset is observed while lateral fluoroscopy shows contrast confined to the annulus or annular–endplate junction, those regions become plausible restorative targets for reparative or biologic strategies aimed at promoting structural healing and reducing peripheral sensitization, rather than simply marking them as surgical levels. A disc that reproduces the presenting pain within the first 2–3 increments is more likely to harbor sensitized annular (and possibly endplate) nociceptors, whereas a disc that tolerates ≈2.0–2.5 mL under these conditions without typical pain is less likely to represent a strongly sensitized annular pain source. These observations should not be interpreted as strict diagnostic cutoffs, but they illustrate how pressure–volume concepts from discometry can be applied pragmatically in routine intradiscal procedures.

This retrospective study has several strengths. It draws on a relatively large cohort from a single practice with a long-standing, standardized discography protocol that enforced strict pressure and volume limits and required concordant pain of at least moderate intensity. A key methodological strength is that, although the present analysis is retrospective, the underlying discography data were prospectively and systematically captured in real time during each procedure using a standardized intra-procedural worksheet and immediate post-procedure dictation into the clinical record, which subsequently fed the electronic database. Quantitative ΔP and work metrics were derived from static plateaus at each 0.5 mL step, and stiffness-normalized checks (K_eq) were used to ensure that onset–censor differences were not simply due to disc compliance. ΔP × work quadrant analyses and within-encounter comparisons further reduced confounding by injector behavior, sedation, and patient-level variability, and device comparisons suggest that the overall pattern is robust across manual and automated injection when strict criteria are applied. Together, these features allow a more detailed characterization of disc responses than is available in most prior series.

The study also has important limitations. It represents the experience of a single, highly specialized center and may not generalize to practices with different patient populations, techniques, or thresholds for calling a disc positive. The analysis is retrospective and relies on construct validity—linking pressure–volume behavior, morphology, and existing histologic data—rather than on direct tissue sampling from the discs in this cohort. Longitudinal outcomes, including surgical or interventional responses, are not addressed in this report, and the subset of discs with Modic changes or predominantly vertebrogenic features is relatively small because of the inclusion criteria used. In addition, the main analysis was limited to levels in which static plateau pressures could be recorded or reliably reconstructed over both low and higher dose ranges (the manual HILO subset). A minority of levels exhibited minimal or absent pressure rise despite incremental volume increases, consistent with possible unrecognized leak pathways through the outer annulus or endplate; these were retained as final-negative (censored) observations because they did not produce concordant pain under bounded stimulation, but such pressure–volume uncoupling may represent a distinct biomechanical subgroup that cannot be fully characterized in this retrospective analysis. This overall selection, while necessary for accurate ΔP and work metrics, may underrepresent levels with incomplete recordings or later procedural variants and thus could introduce subtle selection bias. The step-index analysis was further restricted to a secondary subset of 153 onset-positive levels from 128 encounters, and the resulting volume-staging observations should therefore be viewed as supportive evidence for threshold-like behavior rather than as defining a universal volume cutoff. Disc-level analyses do not fully adjust for clustering of multiple discs within the same patient; our within-encounter paired analyses reduce but do not eliminate this limitation, and the annular versus vertebrogenic interpretation should therefore be viewed as a physiologically grounded model rather than a definitive mechanistic classification for individual motion segments. Sedation during discography was limited to minimal midazolam-based anxiolysis in almost all cases, with very rare use of small doses of meperidine or propofol as described in the Methods; detailed anesthesia flowsheets were not retained, so exact dose distributions cannot be reconstructed, but given the very low doses used any residual influence of sedation on symptom reporting is likely to be small and to bias against, rather than in favor of, detecting low-dose, high-intensity concordant pain. Finally, our earlier pressure-controlled work in asymptomatic volunteers, which included physician discographers among subjects who met asymptomatic criteria, together with subsequent psychometric and systematic review data, suggests that when modern pressure and interpretive criteria are applied, chronic pain status and psychologic scores do not substantially degrade the specificity of discography or inflate false-positive rates, although this conclusion is not universally accepted [14,26,27].

Future work should link pressure–volume metrics and onset thresholds to clinical outcomes, including responses to fusion, motion-preserving procedures, annular- or nucleus-directed biologic therapies, and basivertebral nerve ablation. Prospective studies incorporating standardized imaging, Modic and endplate grading, and, where available, histopathologic or molecular markers of nerve ingrowth and inflammation would help clarify which pressure–volume signatures correspond most reliably to annular, vertebrogenic, or mixed pain generators. Dynamic analysis of intradiscal pressure traces, including temporal patterns and flow-dependent effects, may further distinguish “chemical” low-pressure sensitivity from high-pressure mechanical responses. Ultimately, the goal is not to rehabilitate discography as a universal gatekeeper but to define a limited, physiology-based role for pressure–controlled discography in multimodal evaluation—one that uses quantitative dose–response information to better match patients to the most appropriate tissue-targeted interventions or to conservative care when no clearly nociceptive disc is identified.

## 5 Conclusions

In this retrospective analysis of pressure–volume–controlled discography, structurally abnormal lumbar discs that reproduced the patient's pain (“onset” discs) almost always did so at relatively low static pressure above opening and low cumulative work, yet with moderate-to-severe, clinically robust pain intensity. In contrast, discs that never declared under the same safety caps tolerated higher ΔP and work at their final-negative (censored) steps, and morphologically normal controls occupied the extreme of high-dose tolerance with rare positive responses. These dose–response patterns were consistent across fissured and disrupted discs, within encounters containing both positive and negative discs, and after stiffness normalization, supporting our prespecified hypothesis that onset and censored doses separate in physiologically meaningful ways.

Placed alongside prior mechanical, histologic, and imaging work, these findings are most compatible with a model in which a subset of structurally abnormal discs has become chemically and mechanically sensitized within an overlapping annular–endplate–vertebral nociceptive field, with annular and annular–endplate junctional regions playing a dominant role in low-dose responses. Other discs—whether morphologically normal or with more prominent vertebrogenic features—remain load-tolerant under the bounded pressurization used here. In this framework, pressure-controlled discography functions less as a binary test of “discogenic pain” and more as a probe of dose–response behavior within a shared disc–endplate–vertebral nociceptive zone supplied by sinuvertebral and basivertebral pathways. Low-dose, high-intensity onset in an abnormal disc is best interpreted as reflecting sensitized annular and annular–endplate junctional nociceptors as major contributors, whereas high-dose tolerance despite advanced degeneration or Modic change should prompt greater consideration of vertebrogenic or alternative pain generators.

Our step-index analysis provides a practical extension of these concepts, indicating that in most structurally abnormal discs with a sensitized annular phenotype, concordant pain onset is detectable within a total injected volume on the order of 2 mL under staged, pressure-limited injection (see Section 4.6). This volume-staging behavior suggests that even therapeutic intradiscal injections, when delivered in small increments with brief pauses, can be used to probe for a low-dose annular–junctional phenotype before more invasive or vertebrogenic-targeted interventions are considered.

These conclusions should be viewed as construct validation rather than definitive mechanistic proof and are limited by the retrospective design, single-practice setting, selection of levels with adequate pressure recordings, and lack of longitudinal outcome data. Future prospective work linking pressure–volume signatures to outcomes after annular- and vertebrogenic-targeted interventions and integrating detailed endplate imaging and molecular markers of nerve ingrowth, will be needed to clarify when low-dose responses primarily reflect annular sensitization, when they reflect vertebrogenic mechanisms, and when both contribute meaningfully within the same motion segment. Ultimately, the role of pressure-controlled discography is not to function as a universal gatekeeper but to serve as a selective, physiology-based tool within a multimodal evaluation—providing quantitative dose–response information that helps determine when a disc is likely to be a meaningful nociceptive contributor and when alternative generators or conservative care should be prioritized.

## Declaration of generative AI and AI-assisted technologies in the manuscript preparation process

During the preparation of this work the authors used ChatGPT (OpenAI) to assist with language refinement, organization of existing content, and cross-checking for internal consistency. After using this tool, the authors reviewed and edited the content as needed and take full responsibility for the content of the published article.

## Funding

This research did not receive any specific grant from funding agencies in the public, commercial, or not-for-profit sectors.

## Declaration of competing interest

The authors declare that they have no known competing financial interests or personal relationships that could have influenced the work reported in this paper.

## Appendix A Glossary of key terms and metrics

This glossary summarizes nontrivial terms, operational definitions, and quantitative metrics used in the discometry manuscript. It includes several terms that are specific to this analysis (e.g., “censor,” “onset step index”) and key formulas used in the pressure–volume framework. The glossary is intended as a reference for readers who are not familiar with pressure–volume–controlled discography and to clarify how specific terms are used in this paper.

### Annular nociception

Pain arising primarily from nociceptive fibers located in the intervertebral disc annulus, particularly in the outer annulus and along fissures where nerve ingrowth and sensitization have occurred. In this study, low-dose, high-intensity concordant pain onset in structurally abnormal discs is interpreted as being most consistent with annular-dominant nociception, although vertebral and other contributions may coexist at the same motion segment.

### Basivertebral nerve (BVN)

A nerve arising from the sinuvertebral nerve within the vertebral body that innervates the vertebral endplates and adjacent marrow. BVN-mediated nociception is thought to underlie vertebrogenic pain, especially in the presence of Modic changes and endplate damage.

### Censor (final-negative event)

In this paper, a censor (or final-negative event) refers to the last delivered 0.5 mL step in a disc that never satisfies the predefined onset criteria (definite, sustained concordant pain) under the safety caps of ΔP ≤ 50 psi above opening and total injected volume ≤3.5 mL. The associated static pressure above opening (ΔP_event) and cumulative work (W_event) at this last step are treated as the disc's tolerance dose under these constraints. Censored levels may represent true negatives (discs that do not currently harbor sensitized nociceptors or do not contribute meaningfully to the presenting pain) or false negatives (discs that would declare at higher doses, under different loading conditions, or at another time). The term “censor” is used in the statistical sense to reflect that their pain threshold lies above the observed dose range, not that they are necessarily pain-free at all possible loads.

### Concordant pain onset (onset event)

The first 0.5 mL increment at which the awake patient reports definite, sustained concordant pain—i.e., pain that reproduces their typical symptoms in location and quality, sustained for ≥30 s—under the protocol safety caps (ΔP ≤ 50 psi above opening; total volume ≤3.5 mL). For the primary ΔP and W analyses, onset is operationally defined at the first step where concordant pain reaches at least 4/10 on a 0–10 numeric rating scale, but a small number of discs had onset recorded at intensities <4/10 when the operator judged the match to be clear and later steps confirmed a rise to ≥6/10. The static pressure above opening (ΔP_event) and cumulative work (W_event) at this step are termed the onset dose for that disc.

### ΔP (delta P, static pressure above opening)

Static plateau pressure above opening at a given step, defined as ΔP = P_plateau − P_opening, where P_plateau is the intradiscal pressure (psi) measured after dynamic transients dissipate at that volume, and P_opening is the pressure (psi) at which the nucleus first accepts fluid. In this study, ΔP is used as the primary instantaneous load metric at onset (ΔP_event) and at the final-negative (censored) step. The analysis focuses on ΔP, rather than absolute intradiscal pressure, as a practical surrogate for strain above the disc's intrinsic pressure baseline. The data are consistent with the idea that pain relates more closely to ΔP than to absolute pressure, but they do not prove that the disc “only feels” pressure above opening.

### ΔP × Work quadrants (Q1–Q4)

A 2 × 2 classification of event doses based on static ΔP and cumulative work (W), under prespecified cutoffs. Two grids are used:

• Protocol 20/20 grid: ΔP rungs 10–<20, 20–<30, ≥30 psi; work bands <20, 20–<40, ≥40 psi·mL.
• Historical 15/7.5 grid: ΔP <15 vs ≥ 15 psi; work <7.5 vs ≥ 7.5 psi·mL.

Within each grid, quadrants are defined as: Q1 (low ΔP/low W), Q2 (low ΔP/high W), Q3 (high ΔP/low W), and Q4 (high ΔP/high W). In this study, onset events in structurally abnormal discs were enriched in Q1, whereas censored events clustered in Q4, supporting the interpretation that concordant pain onset is primarily a low-dose phenomenon. For the 15/7.5 grid, the 15 psi cutoff for ΔP reflects historical “chemical disc” thresholds, whereas the 7.5 psi·mL cutoff for work is a conservative value chosen by the authors to represent a reasonably low work dose that is commensurate with the 15 psi ΔP threshold; 7.5 psi·mL is not itself a historical norm but a pragmatic choice to complete the grid.

### Discography (pressure-controlled)

A diagnostic procedure in which contrast is injected into an intervertebral disc while the awake patient reports pain in real time, with intradiscal pressure monitored via a manometer or syringe pump. Pressure-controlled discography refers to techniques in which injection is slow and stepwise, plateau pressures are measured at each increment, and safety caps are enforced (e.g., ΔP ≤ 50 psi above opening; total volume ≤3.5 mL). The present study uses a pressure–volume–controlled protocol (discometry) to quantify static ΔP and work at onset and at censored steps.

### Discometry (pressure–volume–controlled discography)

Operational term used in this paper for discography performed as a dose–response experiment. Infusion begins with a small opening step (≈0.2 mL) followed by fixed 0.5 mL increments, static plateau pressures are recorded after dynamic transients dissipate at each step, and dose is expressed as (1) static ΔP (instantaneous load at the plateau) and (2) mechanical work (W), the cumulative sum of ΔP × ΔV across steps. Discometry treats both positive and negative discs as informative: onset steps provide threshold doses, and censored steps provide tolerance doses under the protocol's safety limits.

### Energy-equivalent stiffness (K_eq)

A stiffness-normalized metric derived from ΔP and work at the event step:

K_eq = 2W / V^2^,

where W is cumulative work to the event (psi·mL) and V is total injected volume (mL) at the event step. Units are psi/mL. K_eq approximates an effective stiffness that accounts for both pressure and volume. In this study, similar median K_eq values for onset and censored events within morphology groups argue against generalized “softness” as the primary explanation for early onset.

### Final-negative dose (censored dose)

The static ΔP and cumulative work (W) measured at the last 0.5 mL step in a disc that does not reach onset criteria under the protocol safety caps (ΔP ≤ 50 psi; total volume ≤3.5 mL). This is treated as the disc's tolerance dose in the analyses. See “Censor (final-negative event)” for interpretive caveats.

### Mechanical work (W)

Cumulative mechanical work performed on the disc up to the event step, approximated as:

W=Σ(ΔP_i×ΔV_i),

where ΔP_i is static pressure above opening (psi) at step i, and ΔV_i is the volume increment (mL) at step i (typically 0.5 mL after the opening step). Units are psi·mL. In this study, W is used as a cumulative “dose” metric alongside instantaneous ΔP.

### Modic changes (MC)

MRI signal changes in the vertebral bone marrow adjacent to the endplates, typically classified as type 1 (edematous/inflammatory), type 2 (fatty), or type 3 (sclerotic). Modic changes are associated with endplate damage and vertebral marrow lesions and are often interpreted as markers of vertebrogenic pain, although they are not specific and not all Modic lesions are symptomatic. In this study, the subset of discs with Modic changes is underrepresented due to inclusion criteria, and the pressure–volume behavior of Modic segments is discussed cautiously as being consistent with vertebrogenic models rather than directly characterized.

### Onset dose (ΔP_event, W_event)

The static ΔP and cumulative work at the onset event:

• ΔP_event: static plateau ΔP at the onset step.
• W_event: cumulative W up to and including the onset step.

These values are compared with corresponding doses at the censored step (final-negative dose) to characterize separation between threshold and tolerance.

### Onset step index

Integer index indicating the 0.5 mL step number at which concordant pain onset occurred, counted after the opening step. Step index 1 corresponds to the first 0.5 mL increment after opening, step index 2 to the second, step index 3 to the third, and step index ≥4 to all later 0.5 mL steps, which are sometimes pooled for descriptive purposes. In this study, onset step indices are used to assess whether pain emerges early (threshold-like) or only after multiple dose increments (gradual “wind-up”). Most onset-positive discs in the analyzed subset declared at step indices 1–2, and approximately 75–80 % of abnormal discs declared by step 3, supporting a threshold-based interpretation under this protocol. A nontrivial but infrequent subset of discs may meet onset criteria at the opening step (onset at opening; see separate entry). Those cases are retained in the main ΔP and W analyses; for the step-index histograms, they are either treated as a special category (step index 0) or excluded from the histogram while still being included in the overall onset dataset. Readers interested in such cases can identify them in the database via the onset_at_opening_flag field.

### Onset at opening (onset_at_opening_flag)

Operational flag indicating discs in which concordant pain onset occurred at the opening step (i.e., when the first small volume of contrast was accepted, before any 0.5 mL increments). These events are infrequent but clinically important, as they imply that even minimal disc distention reproduces the patient's pain. In practice, the operator will almost always deliver at least one additional 0.5 mL increment after the opening step to confirm that the pain response is reproducible and remains concordant, even when initial onset is at opening. In the main analyses of ΔP and W, such discs are retained as onset-positive levels with very low event doses. In step-index plots that index only the 0.5 mL increments, onset-at-opening cases are handled separately (e.g., assigned step index 0 or omitted from the histogram) to avoid distorting the distribution of later step indices. Whether and how to display these cases does not materially affect the primary separation between onset and censored doses but can be explored in sensitivity analyses.

### Opening pressure (P_opening)

The pressure (psi) at which the nucleus first accepts fluid, identified by the initial increase in intradiscal pressure during injection. Opening pressure reflects the disc's baseline mechanical state and posture-dependent load. In this study, pressure metrics are expressed primarily as ΔP above opening rather than absolute pressure.

### HILO parts (hipres/lopres)

In the raw disco_level_parts table, each 0.5 mL injection step is associated with two directly recorded pressure values from the manometer: hipres (the highest pressure observed during that step) and **lopres** (the lowest pressure after approximately 2–4 s of equilibration). These are absolute intradiscal pressures in psi, not adjusted for opening pressure and not expressed as ΔP above opening. In the operative notes they were often documented in shorthand form as “hipres > lopres” (e.g., “25 > 18” in the pressure column for a given step). The term manua**l** HILO subset in this paper refers to the curated set of disc levels in which a majority of steps had reliable hipres/lopres pairs, allowing reconstruction of static plateau pressures and subsequent calculation of ΔP and work. HILO values themselves are not used directly in the ΔP and W metrics, but they provide the underlying high/low pressure data from which static plateaus are derived or validated.

### Positive discogram (operational definition)

In this manuscript, a disc is considered positive if, under the protocol safety limits (ΔP ≤ 50 psi above opening; total volume ≤3.5 mL), the patient reports definite concordant pain that ultimately reaches at least 6/10 in intensity on a 0–10 numeric rating scale and is sustained for ≥30 s, with a clearly concordant match in location and quality. For the primary dose–response analyses, onset is defined at the first step where concordant pain reaches ≥4/10 (see “Concordant pain onset”), but discs are only classified as discogram-positive if later steps confirm intensification to ≥6/10. For some prevalence summaries, a broader category of “any concordant pain” is reported (concordant pain of any intensity), and those cases are not retroactively reclassified in the dose–response grids in this retrospective analysis. As with all discography-based criteria, these operational definitions should be interpreted in conjunction with control discs and the overall clinical context. *See Methods (“Definition of onset and positive discogram”) for full details.*

### Pressure above opening (ΔP above opening)

See “ΔP (delta P, static pressure above opening).” In this paper, ΔP above opening is used as a practical measure of load above the disc's intrinsic baseline. While prior work suggests that ΔP may be more closely related to pain than absolute pressure, the present study interprets ΔP as a physiologically motivated surrogate rather than definitive proof of how discs “sense” load.

### Static vs dynamic pressure

Static pressure refers to plateau intradiscal pressure measured after dynamic transients dissipate at a given volume step; these values are used for ΔP and W calculations. Dynamic pressure refers to transient pressure fluctuations observed during injection or movement of the system; in this study, dynamic traces are used only for quality assurance and not for primary dose metrics.

### Vertebrogenic pain

Pain thought to arise primarily from nociceptive fibers in the vertebral endplate and adjacent marrow, typically carried by the basivertebral nerve. Vertebrogenic pain is associated with endplate microdamage, Modic changes, and vertebral marrow lesions, and may be more dependent on sustained compressive loading and disc–marrow cross talk than on modest, transient intradiscal pressurization. In this study, load-tolerant discs that remain negative despite high ΔP and W, especially in the presence of Modic changes, are discussed as being more consistent with vertebrogenic or mixed disc–vertebral mechanisms, but the dataset is not powered to definitively subtype individual segments.
