# Supplementary material for: Quantitative discometry: Low-dose concordant pain onset identifies sensitized annular nociceptors under pressure–volume–controlled provocation
Source: Interv Pain Med. 2026 Jan 30;5(1):100738. doi: 10.1016/j.inpm.2026.100738 (PMC12874308; doi:10.1016/j.inpm.2026.100738)
Supplement: Multimedia component 1 [file mmc1.docx]

Supplementary Materials

**Table S1. Selection of Disc Levels and Discogram Encounters for the Manual HILO and Main Analytic Cohorts.**

| Step | Selection step | Encounters (n) | Encounters (% of step 1) | Disc levels (n) | Disc levels (% of step 1) |
| --- | --- | --- | --- | --- | --- |
| 1 | All Derby + manual discometry disc levels | 2,183 | 100.0 | 5,812 | 100.0 |
| 2 | Disc levels included in manual hi–lo table | 364 | 16.7 | 859 | 14.8 |
| 3 | Hi–lo levels with ≥70% static pressure–volume pairs | 330 | 15.1 | 698 | 12.0 |
| 4 | Final HILO analytic cohort after all inclusion criteria* | — | — | 477 | 8.2 |
| 5 | Onset-positive levels in HILO analytic cohort† | — | — | 358 | 6.2 |
| 6 | Onset-positive levels with complete intensity data‡ | — | — | 196 | 3.4 |
| 7 | Onset-positive levels with complete step-index data§ | — | — | 153 | 2.6 |

* Final inclusion criteria: lumbar levels, adherence to the ΔP ≤ 50 psi above opening and volume ≤ 3.5 mL protocol caps, adequate morphology/imaging data, and sufficient static plateau coverage (≥70% of recorded steps).

† Onset-positive levels are discs that met the operational onset and discogram-positive criteria (definite concordant pain, sustained ≥30 seconds, onset ≥4/10 with eventual peak ≥6/10) within the protocol caps.

‡ Subset of onset-positive levels with complete pain-intensity ratings (n = 196), representing 41.1% of the analytic cohort (196/477) and 54.7% of onset-positive levels (196/358); used for onset intensity analyses (Section 3.3).

§ Subset of onset-positive levels with complete onset step-index data (n = 153), representing 32.1% of the analytic cohort (153/477) and 42.7% of onset-positive levels (153/358); used for step-index analyses (Supplemental Figure S1).


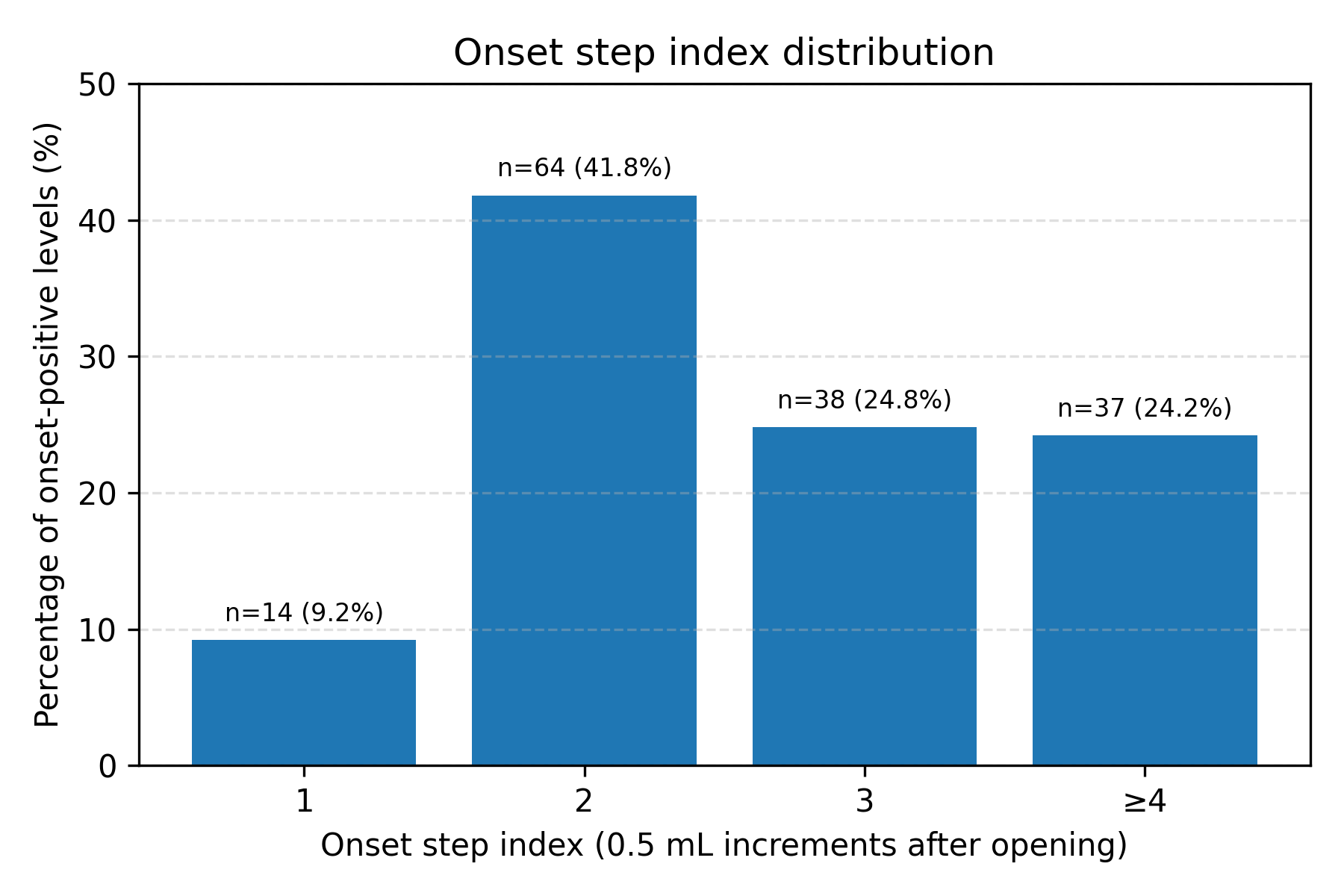

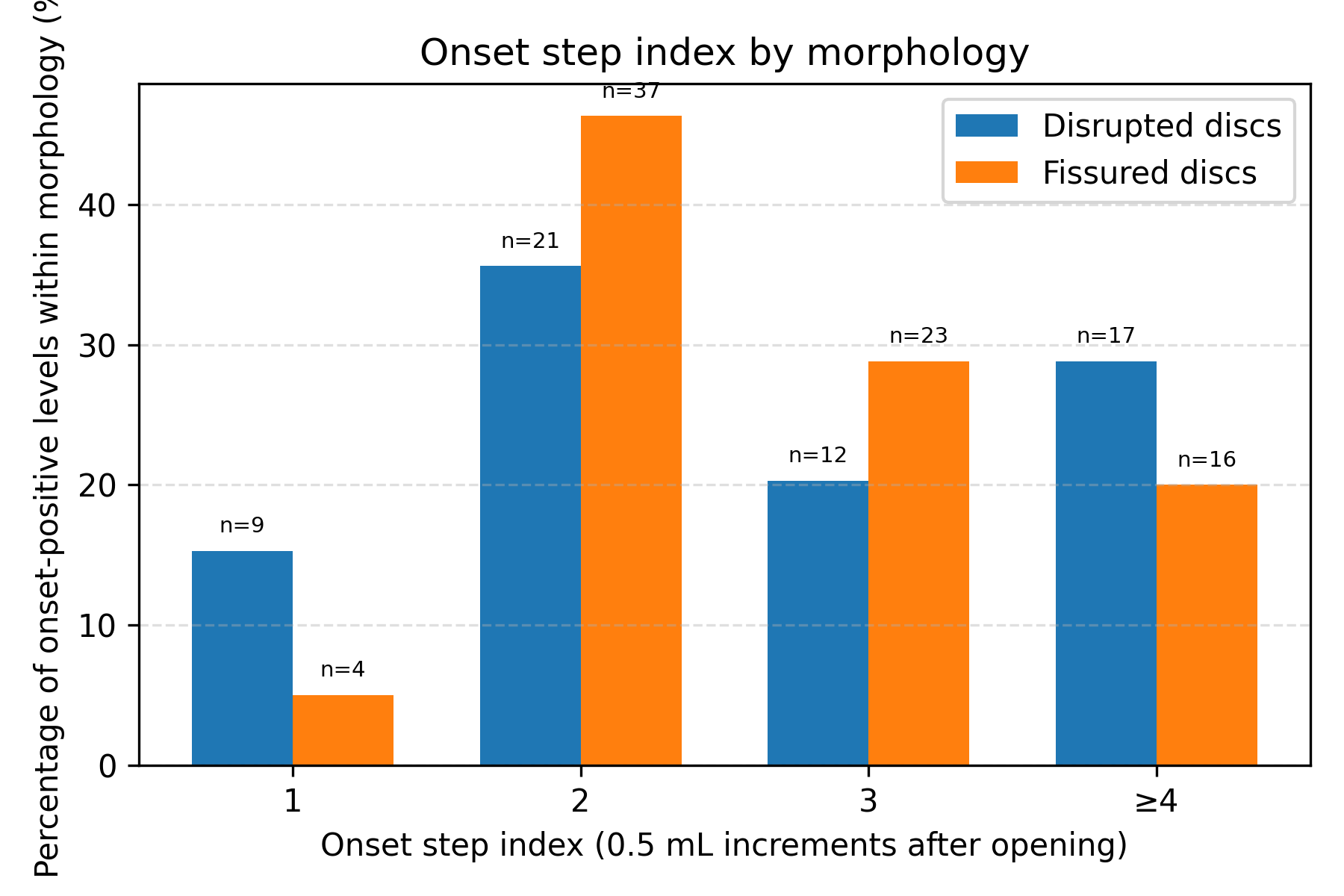


**Supplemental Figure S1. Onset step index under pressure–volume–controlled discography.**

**Panel A (left):** Distribution of the step index at which concordant pain onset occurred among onset-positive disc levels with complete step data (n = 153). Step index 1 denotes the first 0.5 mL increment after the opening step, index 2 the second 0.5 mL increment, index 3 the third, and index ≥4 all later 0.5 mL steps pooled. Bars indicate the percentage of onsets occurring at each index (9.2% at step 1, 41.8% at step 2, 24.8% at step 3, and 24.2% at step ≥4), with labels showing both percentage and count (n = 14, 64, 38, and 37, respectively).

**Panel B (right):** Onset step index distribution stratified by morphology for disrupted (n = 59) and fissured (n = 80) discs. Within disrupted discs, 15.3% of onsets occurred at step 1, 35.6% at step 2, 20.3% at step 3, and 28.8% at step ≥4 (n = 9, 21, 12, and 17, respectively). Within fissured discs, 5.0% of onsets occurred at step 1, 46.3% at step 2, 28.8% at step 3, and 20.0% at step ≥4 (n = 4, 37, 23, and 16, respectively). Both morphologies thus show early declaration concentrated in the first two 0.5 mL steps, with only a minority of discs requiring step index ≥4, supporting a threshold-based interpretation of onset under controlled loading rather than gradual wind-up over many dose increments.

**Supplemental Table S2. Q1 vs Q4 counts for onset and censored events under the protocol 20/20 and historical 15/7.5 grids (overall cohort)**

Table 1

| **Grid (binning scheme)** | **Event type** | **Q1 (low ΔP / low W)** | **Q4 (high ΔP / high W)** | |
| --- | --- | --- | --- | --- |
| Protocol 20/20 grid | Onset events | 180 | 75 |  |
|  | Censored | 106 | 262 |  |
| Historical 15/7.5 grid | Onset events | 105 | 148 |  |
|  | Censored | 43 | 425 |  |

*Counts correspond to the events depicted in Figure 3 and were used for the Q1 vs Q4 odds-ratio calculations reported in Section 3.4. Percentages shown in the text are rounded values derived from these counts.*
